# T Helper Plasticity Is Orchestrated by STAT3, Bcl6, and Blimp-1 Balancing Pathology and Protection in Malaria

**DOI:** 10.1016/j.isci.2020.101310

**Published:** 2020-06-24

**Authors:** Victor H. Carpio, Florentin Aussenac, Lucinda Puebla-Clark, Kyle D. Wilson, Alejandro V. Villarino, Alexander L. Dent, Robin Stephens

**Affiliations:** 1 Department of Microbiology and Immunology, University of Texas Medical Branch, Galveston, TX 77555-0435, USA; 2 Department of Internal Medicine, Division of Infectious Diseases, University of Texas Medical Branch, Galveston, TX 77555-0435, USA; 3 Molecular Immunology and Inflammation Branch, National Institute of Arthritis, Metabolic, and Skin Diseases, National Institutes of Health, Bethesda, MD 20892-1674, USA; 4 Department of Microbiology and Immunology, Indiana University School of Medicine, Indianapolis, IN 46202, USA

**Keywords:** Immunology, Parasitology

## Abstract

Hybrid Th1/Tfh cells (IFN-γ^+^IL-21^+^CXCR5^+^) predominate in response to several persistent infections. In *Plasmodium chabaudi* infection, IFN-γ^+^ T cells control parasitemia, whereas antibody and IL-21^+^Bcl6^+^ T cells effect final clearance, suggesting an evolutionary driver for the hybrid population. We found that CD4-intrinsic Bcl6, Blimp-1, and STAT3 coordinately regulate expression of the Th1 master regulator T-bet, supporting plasticity of CD4 T cells. Bcl6 and Blimp-1 regulate CXCR5 levels, and T-bet, IL-27Rα, and STAT3 modulate cytokines in hybrid Th1/Tfh cells. Infected mice with STAT3 knockout (KO) T cells produced less antibody and more Th1-like IFN-γ^+^IL-21^−^CXCR5^lo^ effector and memory cells and were protected from re-infection. Conversely, T-bet KO mice had reduced Th1-bias upon re-infection and prolonged secondary parasitemia. Therefore, each feature of the CD4 T cell population phenotype is uniquely regulated in this persistent infection, and the cytokine profile of memory T cells can be modified to enhance the effectiveness of the secondary response.

## Highlights

• Plasmodium infection induces a CXCR5^+^IFN-γ^+^IL-21^+^ hybrid Th1/Tfh cell subset
• STAT3/WSX-1, T-bet, Bcl6, and Blimp-1 regulate different aspects of Th1/Tfh phenotype
• T cell-intrinsic STAT3 regulates degree of Th1 commitment of hybrid Th1/Tfh
• Shifting the plastic response toward Th1-like cells promotes resistance from reinfection

## Graphical Abstract

**Figure:**
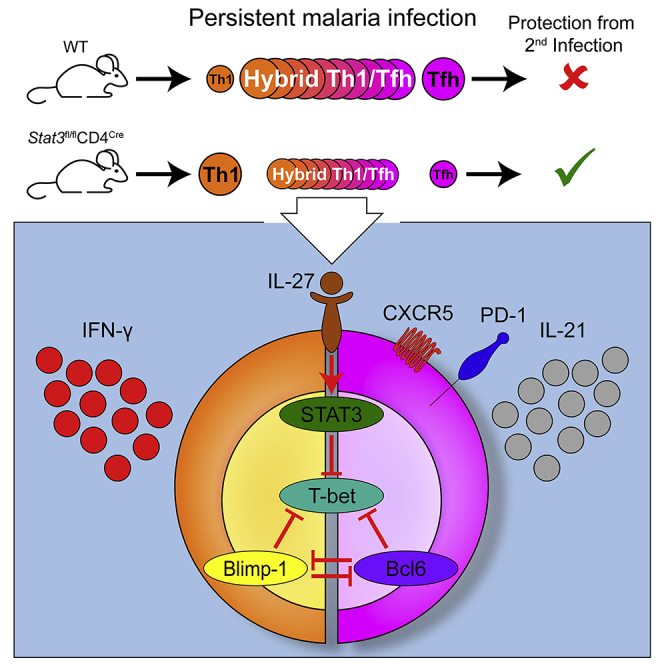

## Introduction

Both cellular and humoral responses are essential for immunity from *Plasmodium* infection. In humans, CD4 T cells that produce interferon (IFN)-γ in response to *Plasmodium falciparum* antigens accumulate with exposure, as do antibodies specific for each variant of parasite the host has been infected with, correlating with lower incidence of both parasitemia and hospitalization. A favorable ratio of interleukin (IL)-10 to tumor necrosis factor (TNF) correlates with resistance from pathology in both mice and people (Li et al., 2003; Luty et al., 1999; May et al., 2000), and CD4 T cells protect immunodeficient mice from dying of *Plasmodium chabaudi* infection (Stephens et al., 2005). Both IL-12 and IFN-γ, T helper-type 1 (Th1)-promoting cytokines, contribute to reduction of peak parasitemia by promoting parasite phagocytosis and generation of Th1-driven antibody isotypes (Su and Stevenson, 2000; Xu et al., 2000). IFN-γ production by T cells in response to *P. chabaudi* infection is initially strong, whereas it becomes downregulated as infection becomes controlled. Thereafter, a much reduced but recrudescent parasitemia is cleared by germinal center (GC)-derived antibody (Perez-Mazliah et al., 2017). IL-21, made predominantly by CXCR5^+^ T cells, including T follicular helper (Tfh), is required for antibody isotype class switch and contributes significantly to full clearance (Carpio et al., 2015; Perez-Mazliah et al., 2015 ).

In *P. chabaudi* infection, we and others have shown that many cells express both IFN-γ and IL-21 (Carpio et al., 2015; Perez-Mazliah et al., 2015). IFN-γ^+^IL-21^+^ CD4 T cells also occur in chronic lymphocytic choriomeningitis virus (LCMV), tuberculosis, and *Listeria* infections (Elsaesser et al., 2009; Li et al., 2016; Tubo et al., 2013). *In vitro*, prolonged T cell receptor (TCR) signaling and IL-12 drive T cells from the Th1 to the Tfh phenotype (Fahey et al., 2011; Schulz et al., 2009; Tubo and Jenkins, 2014). CXCR5^int^ effector T cells (Teff) have been reported in other *Plasmodium* infections and can generate CXCR5^hi^PD-1^hi^ GC Tfh cells in *Plasmodium berghei* (Ryg-Cornejo et al., 2016). Moreover, CXCR5^int^ Teff can help B cells make antibody, although less well than GC Tfh (Obeng-Adjei et al., 2015; Wikenheiser et al., 2018; Zander et al., 2017). We showed that the IFN-γ^+^IL-21^+^CXCR5^+^ T cells in *P. chabaudi* infection express the Tfh markers ICOS and BTLA, along with the IFN-γ-induced chemokine receptor CXCR3, and the primary transcription factors of both Th1 and Tfh (T-bet and Bcl6) (Carpio et al., 2015). These data led us to the term “hybrid Th1/Tfh” to describe any IFN-γ^+^ CD4 T cell also expressing IL-21 and/or CXCR5, functional markers of Tfh. Strikingly, IFN-γ^+^IL-21^+^ T cells are also the main source of IL-10 (Carpio et al., 2015; Perez-Mazliah et al., 2015), a critical cytokine as it prevents lethal pathology in *P. chabaudi*-infected mice (Freitas do Rosario et al., 2012), and promotes antibody responses (Guthmiller et al., 2017). Hybrid Th1/Tfh cells also preferentially expand during *P. falciparum* infection, where they have been termed Th1-like Tfh (Obeng-Adjei et al., 2015). However, Bcl6-deficient T cells adoptively transferred into wild-type (WT) mice differentiated into both CXCR5^int^ and IFN-γ^+^IL-21^+^ T cells in *P. chabaudi* infection (Carpio et al., 2015), suggesting that these hybrid phenotype T cells are not of the Tfh lineage. The impaired ability of hybrid Th1/Tfh to help antibody production is likely due to an antagonism regulating Tfh effector functions through the network of STAT4 and T-bet expression and the effects of IL-2, IL-12, IFN-γ, and/or TNF, depending on the infection (Fang et al., 2018; Weinstein et al., 2018). In *P. berghei* ANKA infection, IFN-γ and/or TNF and T cell-intrinsic T-bet inhibit GC Tfh, GC B cell formation, and IgG production in response to infection (Ryg-Cornejo et al., 2016). Therefore, the hybrid Th1/Tfh population producing IFN-γ, IL-21, and IL-10 are likely to concurrently provide cellular protection and limit the large humoral response, which leads to hypergammaglobulinemia. It is not well understood which differentiation pathways control expression of these effector cytokines, particularly in persistent infections. Therefore, we have investigated the molecular regulation of T cell cytokine production and phenotype in response to infection with *Plasmodium spp.* through T cell-specific genetic manipulation to the test the importance of Th differentiation and plasticity *in vivo*.

Classically, committed IFN-γ^+^ Th1 cells are generated by antigen stimulation in the presence of IL-12, which signals through STAT4 (Hsieh et al., 1993) which maximizes levels of the master regulator of Th1 differentiation, T-bet (Szabo et al., 2000). Th1 cells express CXCR3, but not CXCR5, which allows them to migrate away from the B cell follicle into the red pulp and inflamed tissues. Fully differentiated GC Tfh cells are identified as CXCR5^hi^PD-1^hi^, and their generation depends on the Tfh cell lineage-determinant transcription factor Bcl6 (Johnston et al., 2009; Nurieva et al., 2009). Many cytokines that regulate Tfh development, including IL-6, IL-27, and IL-21, signal through STAT3 (Crotty, 2014). IL-6 signaling through STAT3 secures Tfh programming by limiting Th1 differentiation (Choi et al., 2013). IL-27 signaling through STAT3 induces IL-21 production in T cells (Batten et al., 2010), which in turn promotes Tfh development (Nurieva et al., 2008). *In vitro* and in response to viral infection, STAT3-deficient T cells have a defect in Tfh differentiation (Ray et al., 2014), whereas humans with STAT3 dominant-negative mutations have compromised Tfh development (Ma et al., 2012). However, over the last few years, several lines of evidence suggest a complex regulation of Th1 and Tfh, where lineage determination is intertwined at the molecular level (Weinmann, 2014). For example, the transcription factor Blimp-1 can inhibit both Tfh and Th1 differentiation via transcriptional inhibition of Bcl6 and T-bet, respectively (Cimmino et al., 2008; Johnston et al., 2009). In the context of persistent infection, Blimp-1 also controls IL-10 production by Th1 cells (Parish et al., 2014). Therefore, we used an *in vivo* approach involving the most relevant transcription factors reported to date to understand the molecular regulation of T cells and protective responses to *Plasmodium spp.* infections.

Both Th1 and Tfh responses are critical for malaria immunity; however, the ideal balance between these T cell subsets remains unclear. Therefore, we investigated the roles of STAT3, T-bet, Bcl6, and Blimp-1 in the development of hybrid Th1/Tfh cells during persistent *P. chabaudi* infection to identify protective responses. We found that in contrast to the hybrid Th1/Tfh cells found in WT mice upon infection, T cells from T cell-specific STAT3-deficient mice (*Stat3*^fl/fl^CD4^Cre^, STAT3 TKO) preferentially differentiated into Th1 memory cells (IFN-γ^+^IL-21^−^T-bet^hi^). Strikingly, STAT3 TKO mice were 100% protected from reinfection, whereas T-bet-deficient mice had no Th1 memory cells and higher parasitemia. Both mice had reduced serum levels of *Plasmodium*-specific IgG2b, the Th1 isotype, suggesting that the strong positive effect on parasitemia in STAT3 TKO mice was due to improved Th1 memory. Mechanistically, T-bet, and not STAT1 or STAT4, regulated IFN-γ production by T cells; and T cell-intrinsic expression of STAT3, Bcl6, and Blimp-1 each regulated T-bet expression during the peak of infection. Therefore, STAT3 is a key player regulating protection and the cytokine plasticity of memory T cells in malaria. These data support the hypothesis that Th cell pluripotency allows continued responsiveness promoting control of persistent infections and host homeostasis.

## Results

### *Plasmodium* Infections Induce Hybrid Th1/Tfh and GC Tfh Cells

We have previously reported the presence of hybrid Th1/Tfh cells expressing both Tfh markers (CXCR5, ICOS, BTLA, IL-21, and Bcl6) and Th1 markers (CXCR3, IFN-γ, T-bet), as well as the regulatory cytokine IL-10 within effector T cells (CD4^+^CD44^hi^CD127^-^) in *P. chabaudi* infection on day 7 post-infection (p.i.) (Carpio et al., 2015). Although hybrid Th1/Tfh cells have been described in several infections, including LCMV Clone 13 and tuberculosis, the timing of their generation has not been investigated to date. It is important to distinguish the hybrid Teff from GC Tfh, which are essential for GC formation. Therefore, we infected C57BL/6J mice with *P. chabaudi* (AS) or *P. yoelii* (17XNL) infected red blood cells (iRBCs, Figure S1A) and measured parasitemia. Using flow cytometry, we measured GC B cell numbers and the expression of CXCR5, PD-1, IFN-γ, IL-21, T-bet, and Bcl6 in Teff for the first 30 days of infection. We identified Th1-like cells as positive for only Th1 markers (IFN-γ^+^and/or T-bet^+^, but IL-21^−^CXCR5^-^), Tfh-like cells as positive for only Tfh markers (IL-21^+^CXCR5^+/int^, but IFN-γ^-^and/or T-bet^-^), and hybrid Th1/Tfh as cells that express any Th1, along with any Tfh marker. GC Tfh have been defined in the literature as (CXCR5^hi^PD-1^hi^), and we follow that convention throughout. GC B cells (B220^+^GL-7^+^CD38^lo^) are highly visible in the third week of the response to both species. Unbiased t-distributed stochastic neighbor embedding analysis gated on CD4^+^ T cells (Figure S1B) shows the small cluster of GC Tfh (Bcl6^hi^CXCR5^hi^PD-1^hi^) and the larger islands of hybrid Th1/Tfh cells (IFN-γ^+^IL-21^+^) generated in response to both infections.

**Figure 1:**
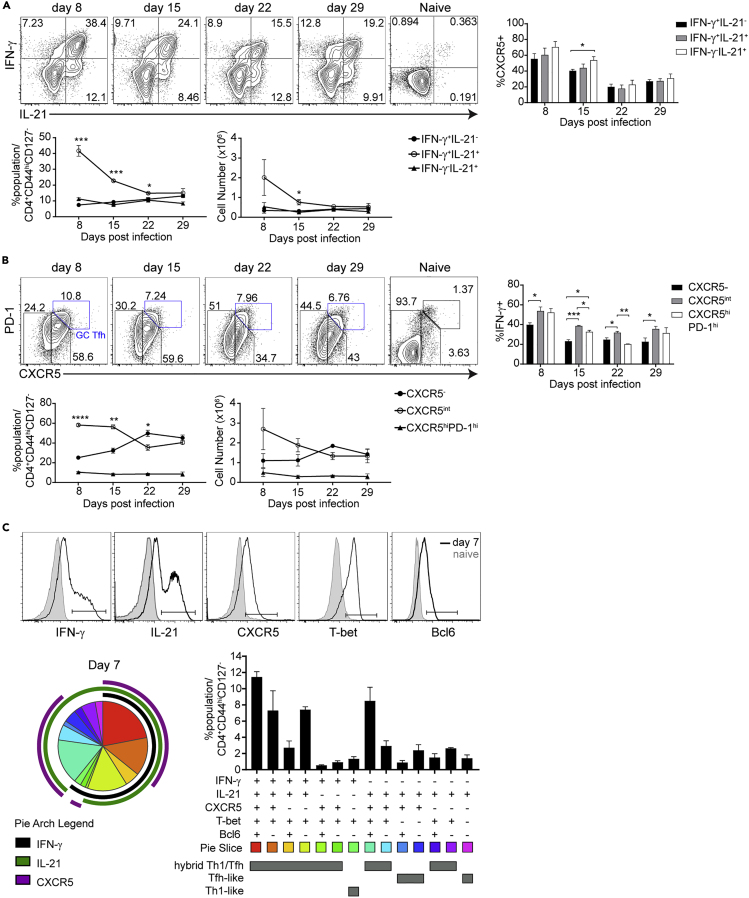
T Helper Differentiation during *P. chabaudi* Infection Resembles a Hybrid Th1/Tfh Phenotype C57BL/6J mice were infected with *P. chabaudi* (10^5^ iRBCs), and splenocytes were analyzed on the days post-infection indicated. (A) Expression of IFN-γ and IL-21. Below, line graphs show percentage (left) and numbers (right) of IFN-γ^+^IL-21^-^ (black filled dots), IFN-γ^+^IL-21^+^ (open circles), and IFN-γ^-^IL-21^+^ (filled triangles) Teff. Bar graph on the right shows CXCR5 expression in cytokine-producing populations. (B) Expression of PD-1 and CXCR5 on CD4 Teff (CD44^hi^CD127^-^) and naive (CD44^lo^CD127^+^). Below, line graphs show percentage (left) and numbers (right) of CXCR5^-^ (black filled dots), CXCR5^int^ (open circles) Teff, and CXCR5^hi^PD-1^hi^ GC Tfh (filled triangles) populations. Bar graph on the right shows IFN-γ production by the different populations. (C) Boolean gating of all possible combinations of IFN-γ, IL-21, CXCR5, T-bet, and Bcl6 expression of CD4 Teff on day 7 p.i. Top, histograms show gate marker used to define IFN-γ^+^, IL-21^+^, CXCR5^+^, T-bet^+^, and Bcl6^+^ in CD4 Teff. Bottom left, pie chart shows the distribution of subsets. Bottom right, bar graph shows the percentage of the subsets. Data representative of 3 experiments with 3 mice/group. Data are represented as mean ± SEM. ^∗^ p≤0.05, ^∗∗^ p≤0.001, ^∗∗∗^ p≤0.0001, ^∗∗∗∗^ p≤0.0001. See also Figure S1.

Throughout, we identify Teff as CD44^hi^CD127^-^, as IL-7Rα (CD127) is transiently downregulated upon activation, with negative expression in Teff on day 9 p.i. (Stephens and Langhorne, 2010). CD127 downregulation correlates with CD11a high expression (Figure S1C), which is upregulated by TCR, but not cytokine stimulation (McDermott and Varga, 2011), suggesting that CD127^-^ is also a marker of TCR stimulation. In the first week of infection, the majority of Teff produce both IFN-γ and IL-21, averaging 41.67% of Teff in *P. chabaudi* and 48.57% in *P. yoelii* in the first week of infection (Figures 1A and S1D). The IFN-γ^+^IL-21^+^ Teff population decreases by half in the second week and then has a stable presence. In addition, there is an increase of CXCR5 expression on Teff in response to both *P. chabaudi* and *P. yoelii* (Figures 1B and S1E). GC Tfh cells are present in stable numbers starting in the first week in both infections, as previously suggested (Wikenheiser et al., 2016). Boolean gating analysis using IFN-γ, IL-21, CXCR5, T-bet, and Bcl6 at day 7 p.i. showed that 66.43% Teff from *P. chabaudi*-infected mice co-express IFN-γ^+^ and at least one marker of Tfh (IL-21, Bcl6, or CXCR5), with IL-21^+^IFN-γ^+^ included in the majority of those sub-populations (Figures 1C and S1F). The other population represented at over 4% of Teff is positive for all markers, including T-bet, but not IFN-γ. On the other hand, the IFN-γ^+^T-bet^+^ Th1-like cells represent a modest fraction (2.91%) of the response. Therefore, infection with *Plasmodium spp.* drives generation of a large population of IFN-γ^+^IL-21^+^ hybrid Th1/Tfh effector cells, which peak in the first week, as well as GC Tfh that are more stably represented, but very few IFN-γ^+^ Th1-like cells without Tfh markers. These IFN-γ^+^IL-21^+^ hybrid Th1/Tfh cells are reminiscent of CD4 T cells identified in other persistent infections (Crawford et al., 2014), leading us to investigate the role of continuing infection in their generation, and to identify molecular mechanisms regulating their generation.

### Shorter Infection Results in Fewer Hybrid Th1/Tfh Cells

**Figure 2:**
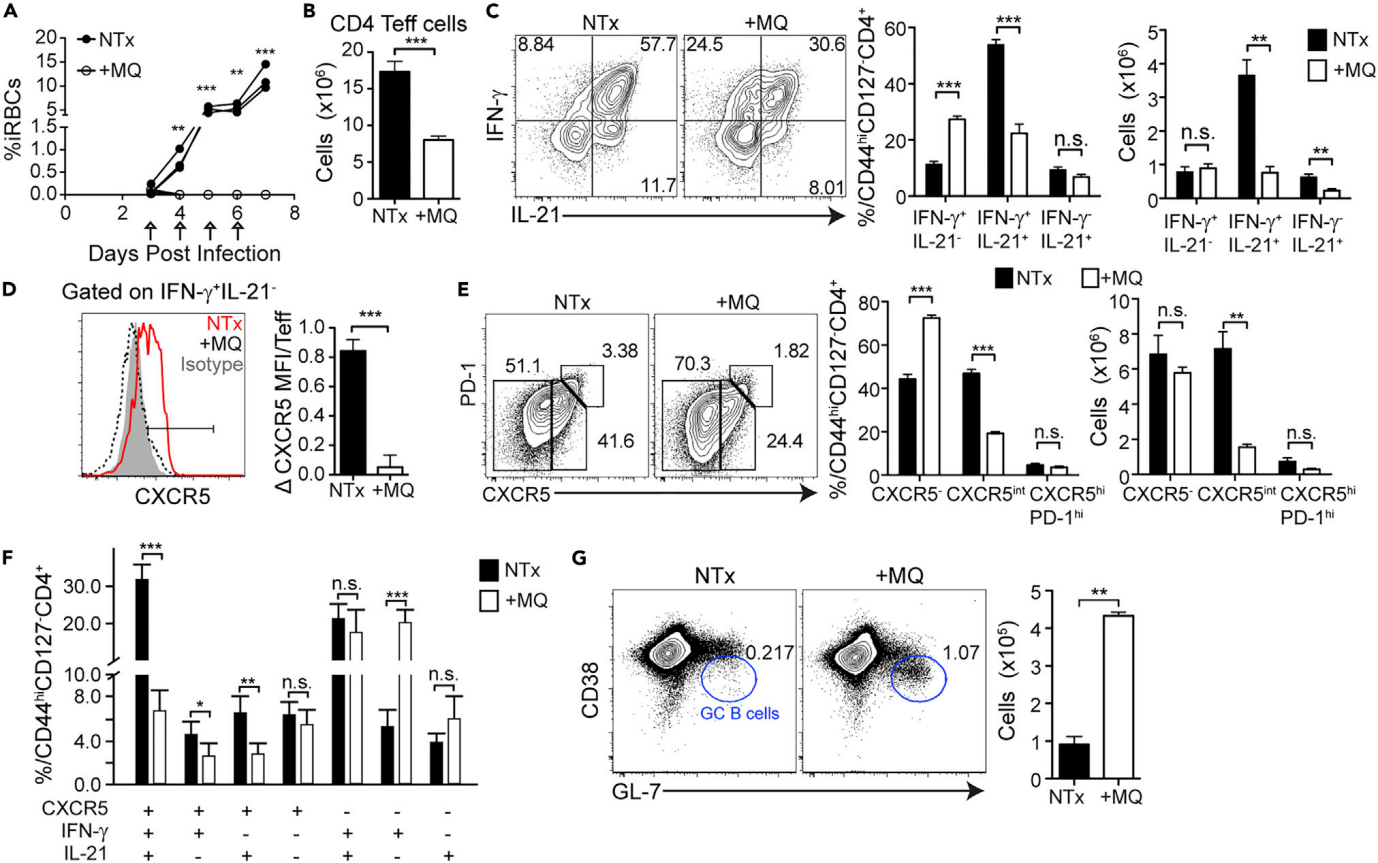
Drug-Cured Mice Have Fewer IFN-γ^+^IL-21^+^CXCR5^+^ Hybrid Th1/Tfh and More IFN-γ^+^IL-21^−^CXCR5^-^ Th1-Like Cells C57BL/6J mice were infected, one group was treated with anti-malarial drug mefloquine (MQ) by oral gavage starting day 3 p.i. and splenocytes analyzed at day 7 p.i. (A) Parasitemia in treated (+MQ, open circles) and not-treated (NTx, black filled circles) groups. Arrows indicate MQ treatment. (B, C, and E) (B) CD4 Teff numbers in +MQ (white bar) and NTx (black bar). (C) Expression of IFN-γ and IL-21 and (E) PD-1 and CXCR5 in Teff. Bar graphs show percentages and numbers of Teff subsets. (D) Histogram overlay shows CXCR5 expression in Th1-like IFN-γ^+^IL-21^-^ Teff. Bar graph shows fold change of CXCR5 mean fluorescence intensity (MFI) over isotype control. (F) Boolean gating analysis of CXCR5^+^, IFN-γ^+^, and IL-21^+^ expression by Teff. (G) Expression of CD38 and GL-7 on B cells (gated on B220^+^MHCII^+^). Bar graph shows numbers of GC B cells (CD38^lo^GL7^+^) per spleen. Data representative of 3 experiments with 3–4 mice/group. Data are represented as mean ± SEM. ^∗^ p <0.05, ^∗∗^ p <0.01, ^∗∗∗^ p <0.001, ^∗∗∗∗^ p <0.0001, n.s., not significant. See also Figure S2.

Hybrid Th1/Tfh cells have been documented in human patients with malaria (Obeng-Adjei et al., 2015) and in other persistent infections including LCMV Clone 13 (Crawford et al., 2014; Nakayamada et al., 2011) using various combinations of Th1 and Tfh markers. On the other hand, acute infections can promote independent differentiation of Th1 and Tfh populations (Curtis et al., 2010; Hale et al., 2013). We have previously shown that complete parasite clearance by the antimalarial drug mefloquine (MQ) given starting on day 3 p.i. increased the Tcm/Tem ratio in the memory phase compared with persistently infected animals (Opata et al., 2015). As no qualitative change in phenotype was observed when drug treatment began on day 5 or 30 p.i., there seems to be a limited window for determining the quality of T cell priming. As Tcm and Tfh generation seems to be linked (Pepper et al., 2011), we tested if limiting the duration of infection by drug treatment would alter the T cell cytokine profile away from IFN-γ^+^IL-21^+^ hybrid Th1/Tfh. MQ treatment of *P. chabaudi*-infected animals starting on day 3 cleared infection almost completely by day 5 (Figure 2A). MQ treatment has no known effect on immune cells at this low dose (Paivandy et al., 2014). Stopping the infection early (+MQ) decreased the numbers of Teff (Figure 2B). In addition, there were also striking qualitative changes. MQ-treated animals had a higher fraction of Th1-like IFN-γ^+^IL-21^-^ Teff, and a strong reduction in the fraction and number of IFN-γ^+^IL-21^+^ T cells (Figure 2C). These IFN-γ^+^IL-21^-^ Teff also did not express more CXCR5 than naive T cells, unlike the Th1-like cells in persistent *P. chabaudi* (Figure 2D). In fact, treatment of infection significantly reduced the proportions of all CXCR5^int^ cells in the Teff population at day 7 p.i. (Figure 2E). Examining all markers together, MQ treatment reduced the proportion of IFN-γ^+^IL-21^+^CXCR5^+^ by 79.33% ± 3.41%, but not IFN-γ^+^IL-21^+^CXCR5^-^, and increased the proportions of Th1-like IFN-γ^+^CXCR5^−^IL-21^-^ compared with untreated animals (Figure 2F), suggesting that generation of CXCR5^-^ Th1-like cells is inhibited by infection lasting longer than 3 days. To investigate any potential role of hybrid Th1/Tfh in early GC formation, we measured GC B cells on day 7, the day of peak T-bet expression in T cells. GC B cell numbers were increased at day 7 p.i. in treated compared with untreated mice (Figure 2G), opposite to hybrid Th1/Tfh cells. The untreated mice also showed a distinct population of CD38^hi^GL-7^+^ B cells, which has been previously described as GC-independent memory B cell precursors (Taylor et al., 2012). In contrast, starting treatment on day 5 rather than day 3 reduced infection immediately (Figure S2A), but had no effect on the fraction of IFN-γ^+^IL-21^+^ (Figure S2B) or CXCR5^int^ Teff (Figure S2C). T cell priming occurs before day 5 of *P. chabaudi* infection (Opata et al., 2015; Sponaas et al., 2012). Therefore, we conclude that the cytokine milieu surrounding antigen presentation regulates priming of the hybrid Th1/Tfh cell phenotype. However, the transcriptional mechanisms regulating this new phenotype are not clear.

### T-bet Regulates IFN-γ and IL-21 Production by Hybrid Th1/Tfh Cells

**Figure 3:**
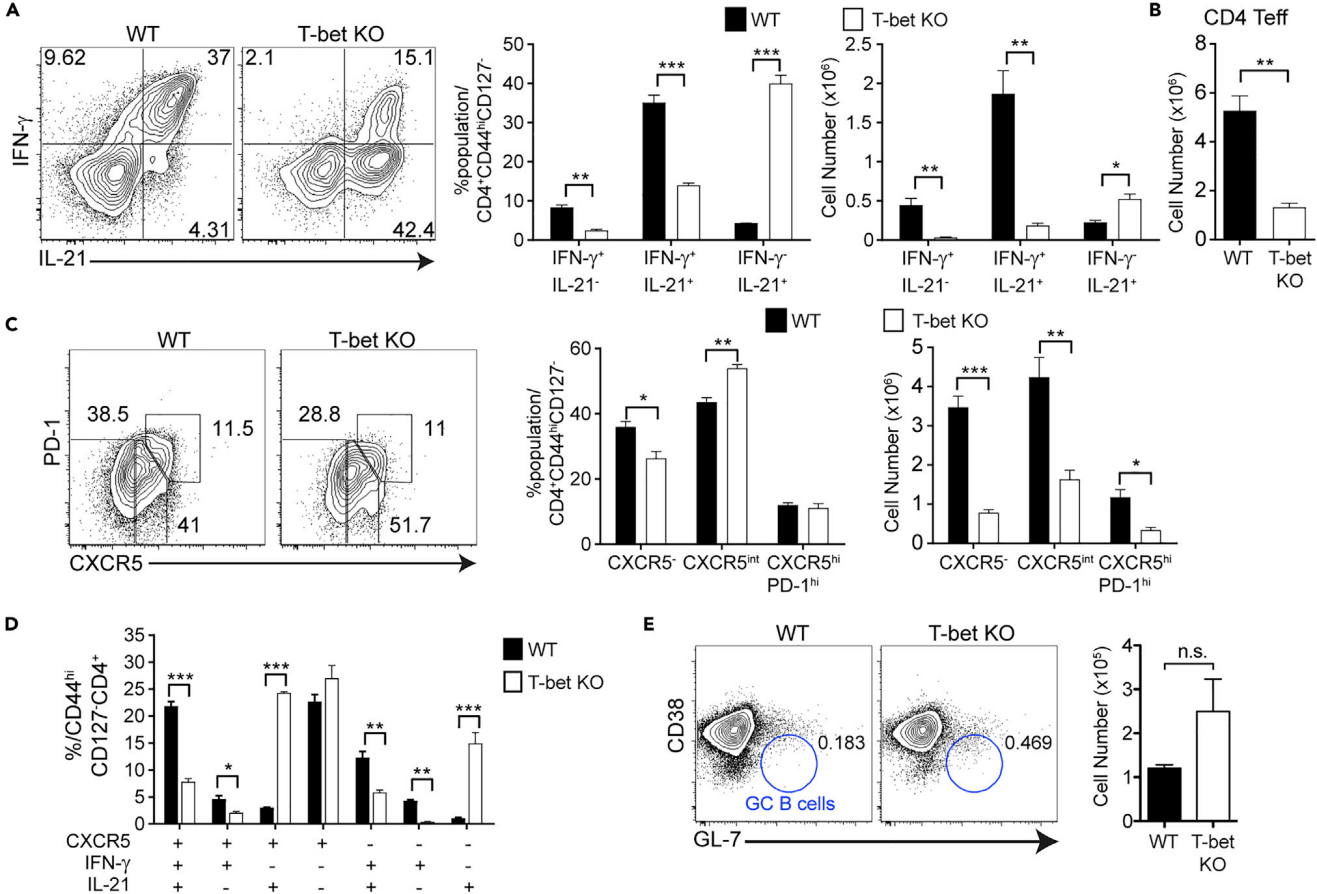
T-bet Deficiency Reduces IFN-γ^+^IL-21^+^CXCR5^+^ Hybrid Teff and Th1 but Promotes IFN-γ^-^IL-21^+^ T Helper Differentiation in *P. chabaudi* Infection T-bet KO and WT animals were infected, and splenocytes were analyzed at day 8 p.i. (A) Expression of IFN-γ and IL-21 in Teff. Bar graphs show percentages and numbers in WT (black bars) and T-bet KO (white bars). (B) Bar graph shows CD4 Teff numbers. (C and D) (C) Expression of PD-1 and CXCR5 in Teff. (D) Boolean analysis of CXCR5^+^, IFN-γ^+^, and IL-21^+^ within WT and T-bet KO Teff. (E) CD38 and GL-7 in B cells (B220^+^MHCII^+^). Bar graph shows numbers of GC B cells (CD38^lo^GL7^+^). Data representative of 2 experiments, 3–8 mice/group. Data are represented as mean ± SEM. ^∗^ p <0.05, ^∗∗^ p <0.01, ^∗∗∗^ p <0.001, ^∗∗∗∗^ p >0.05, n.s, not significant. See also Figure S3.

Th1 cells play a crucial role in immunopathogenesis and host survival in *Plasmodium spp.* infection (Oakley et al., 2013; Su and Stevenson, 2000). Basal levels of T-bet expression can be driven by TCR signaling, IFN-γ, and STAT1. T-bet then upregulates IL-12Rβ2, promoting IL-12 signaling through STAT4, to drive increased T-bet expression and full Th1 commitment (Afkarian et al., 2002; Szabo et al., 2000). Interestingly, T-bet has been shown to work in concert with Bcl6 to regulate the plasticity of Th1 cells (Oestreich et al., 2012). Although we have observed very few T-bet^hi^ Th1 committed cells in our studies, most Teff express T-bet at a low level (Carpio et al., 2015). The role of Th1 transcriptional activators in *P. chabaudi* infection has not been well established, particularly in the differentiation of hybrid Th1/Tfh cells. Using *P. chabaudi*-infected mutant mice, we found that STAT4 was not required for the generation of IFN-γ^+^IL-21^+^ Teff (Figure S3A), but it was critical for GC Tfh differentiation (Figure S3B). Although surprising, this agrees with recent reports showing a role for STAT4 in generating GC Tfh in infection (Weinstein et al., 2018). T cell-intrinsic STAT1 was not required for generation of IFN-γ^+^IL-21^+^ T cells as well (Figure S3C). T-bet-deficient (*tbx21*^-/-^, T-bet knockout (KO)) mice infected with *P. chabaudi* had a strong reduction in IFN-γ production and a significant increase in the fraction and number of IL-21^+^IFN-γ^-^ Tfh-like cells (Figure 3A). The overall percentage of IL-21^+^ Teff in WT mice was 38.33% ± 1.77%, whereas in KO mice was 52.93% ± 2.38% (*p* = 0.008), suggesting a role for T-bet in IL-21 production. In addition, there was a large decrease in the overall numbers of *tbx21*^-/-^ Teff compared with WT on day 7 p.i. (Figure 3B). T-bet deficiency increased the level of expression of CXCR5 on Teff but had no effect on the relative fraction of GC Tfh (Figure 3C). Boolean gating analysis revealed a reduction in hybrid IFN-γ^+^IL-21^+^CXCR5^+^ cells and a shift toward more Tfh-like Teff (IFN-γ^-^IL-21^+^CXCR5^+/−^) in the absence of T-bet (Figure 3D). However, we did not identify a significant change in the number of GC B cells at day 7 p.i. (Figure 3E). Supporting an important role for IFN-γ^+^ Teff and T-bet^+^ B cells in control of this infection, 40% of T-bet KO mice died from infection (Figure S3D). T-bet KO mice that survived the infection did not control parasitemia as well as WT (Figure S3E) and had worse weight loss and hypothermia (Figure S3F). These data suggest that T-bet regulates IFN-γ and IL-21 production by hybrid Th1/Tfh cells. Moreover, T-bet expression is required for control of parasitemia and immunopathology in *P. chabaudi* infection.

### Bcl6 and Blimp-1 Regulate CXCR5 Levels in *P. chabaudi* Infection

**Figure 4:**
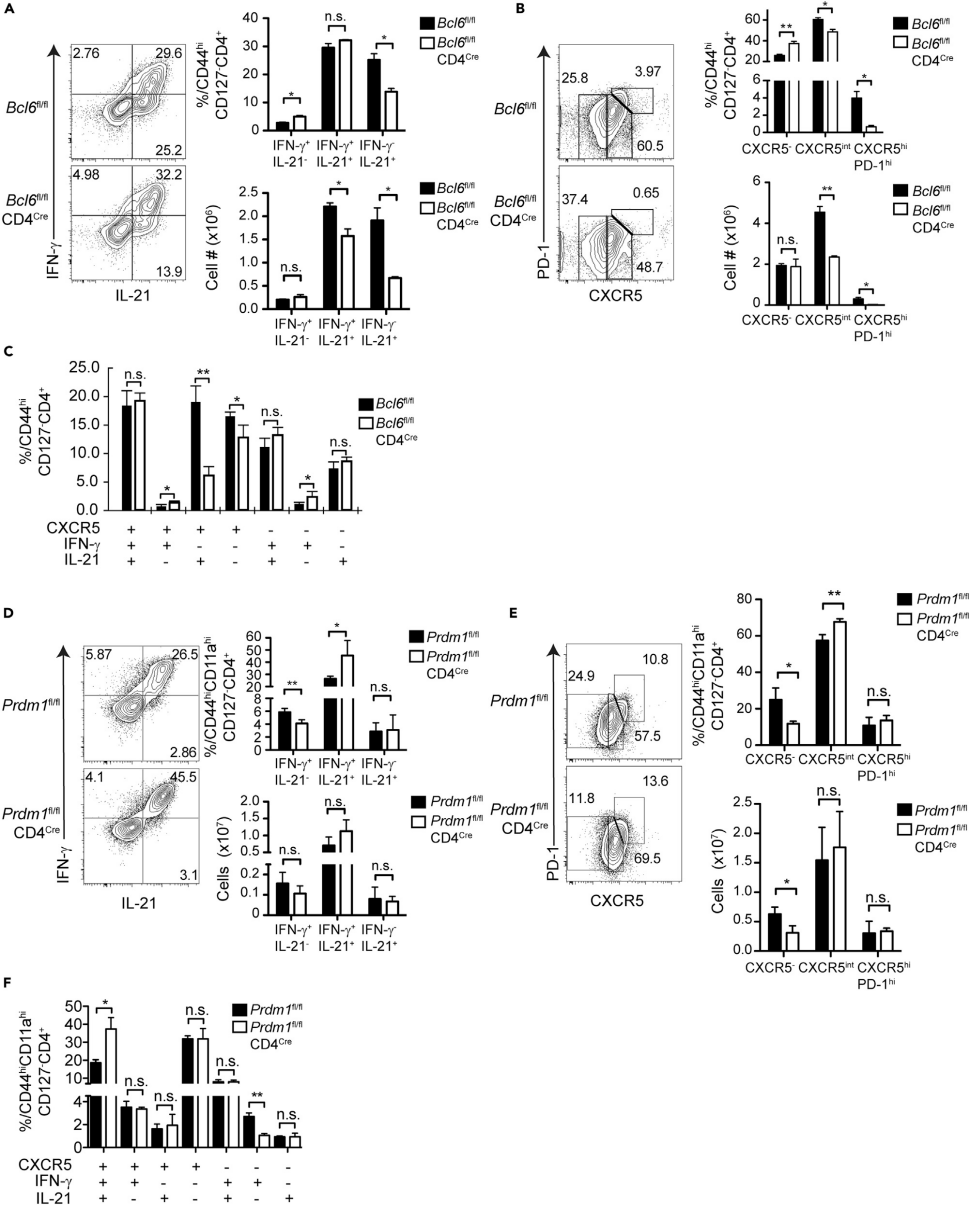
Roles of Bcl6 and Blimp in T Cell Differentiation during *P. chabaudi* Infection (A–C) *Bcl6*^fl/fl^CD4^Cre^ (TKO) and *Bcl6*^fl/fl^ (WT) animals were infected, and splenocytes were analyzed at day 7 p.i. Contour plots and bar graphs show expression of (A) IFN-γ/IL-21 or (B) PD-1/CXCR5 gated on Teff. (C) Boolean analysis of CXCR5^+^, IFN-γ^+^, and IL-21^+^ subsets within WT (black bar) and Bcl6 TKO (white bar) Teff. (D–F) *Prdm11*^fl/fl^CD4^Cre^ (Blimp-1 TKO) and *Prdm11*^fl/fl^ (WT) animals were infected and splenocytes were analyzed at day 7 p.i. Contour plots and bar graphs show subsets of (D) IFN-γ/IL-21 or (E) PD-1/CXCR5 gated on Teff. (F) Boolean analysis of CXCR5^+^, IFN-γ^+^, and IL-21^+^ within WT (black bars) and Blimp-1 TKO (white bars) Teff. Data representative of 3 experiments, 3–4 mice/group. Data are represented as mean ± SEM. ^∗^ p <0.05, ^∗∗^ p <0.01, n.s. p >0.05, not significant. See also Figure S4.

The major Tfh regulatory transcription factor, Bcl6 can bind T-bet and inhibit its function (Oestreich et al., 2012), and indeed, we previously reported that Bcl6 levels correlate with the level of *ifng* transcription in Teff in *P. chabaudi* (Carpio et al., 2015). To test the role of Bcl6 in the differentiation of hybrid Th1/Tfh, we infected *Bcl6*^fl/fl^CD4^Cre^ (Bcl6 TKO) and *Bcl6*^fl/fl^ (WT) mice with *P. chabaudi*. The percentage of IFN-γ^+^IL-21^+^ Teff did not change in the Bcl6 TKO mice on day 7 p.i, although IFN-γ^-^IL-21^+^ and overall Teff numbers were reduced (Figure 4A). As expected, *bcl6*^−/−^ Teff did not generate GC Tfh (Figure 4B). Interestingly, the proportion and numbers of CXCR5^+^ Teff decreased at day 7 p.i. on *bcl6*^−/−^ Teff. Overall, Bcl6 deficiency resulted in an average 65% reduction of CXCR5^+^IL-21^+^IFN-γ^-^ Tfh-like fraction (Figure 4C). We confirmed that T cell-specific Bcl6 deficiency had an effect only on parasite clearance (Figure S4A, Perez-Mazliah et al., 2017) and a slight increase in IL-10 in T cells (data not shown).

In CD4 T cells, Blimp-1 can inhibit both T-bet and Bcl6 and is known to promote IL-10 production in *P. chabaudi* (Cimmino et al., 2008; Montes de Oca et al., 2016). We also tested the role of Blimp-1, the reciprocal regulator of Bcl6 (Johnston et al., 2009), infecting *Prdm1*^fl/fl^CD4^Cre^ (Blimp-1 TKO) animals. We found modest differences in cytokine production (Figure 4D). However, the percentage, but not the number, of CXCR5^+^ Teff was increased in *prdm1*^−/−^ Teff due to a shift in mean fluorescence intensity (Figure 4E). Boolean analysis revealed that the relative fraction of IFN-γ^+^IL-21^+^CXCR5^+^ hybrid Th1/Tfh was also increased, whereas Th1 IFN-γ^+^IL-21^−^CXCR5^-^ cells decreased in infected Blimp-1 TKO animals (Figure 4F). Despite equal parasite levels, all the Blimp-1 TKO mice died, similar to IL-10 KO mice (Figure S4B). In summary, Bcl6 and Blimp-1 coordinately regulate CXCR5 (and IL-10) expression levels in hybrid Th1/Tfh cells.

### Effector T Cells Deficient in STAT3 Become More Th1-like Cells

**Figure 5:**
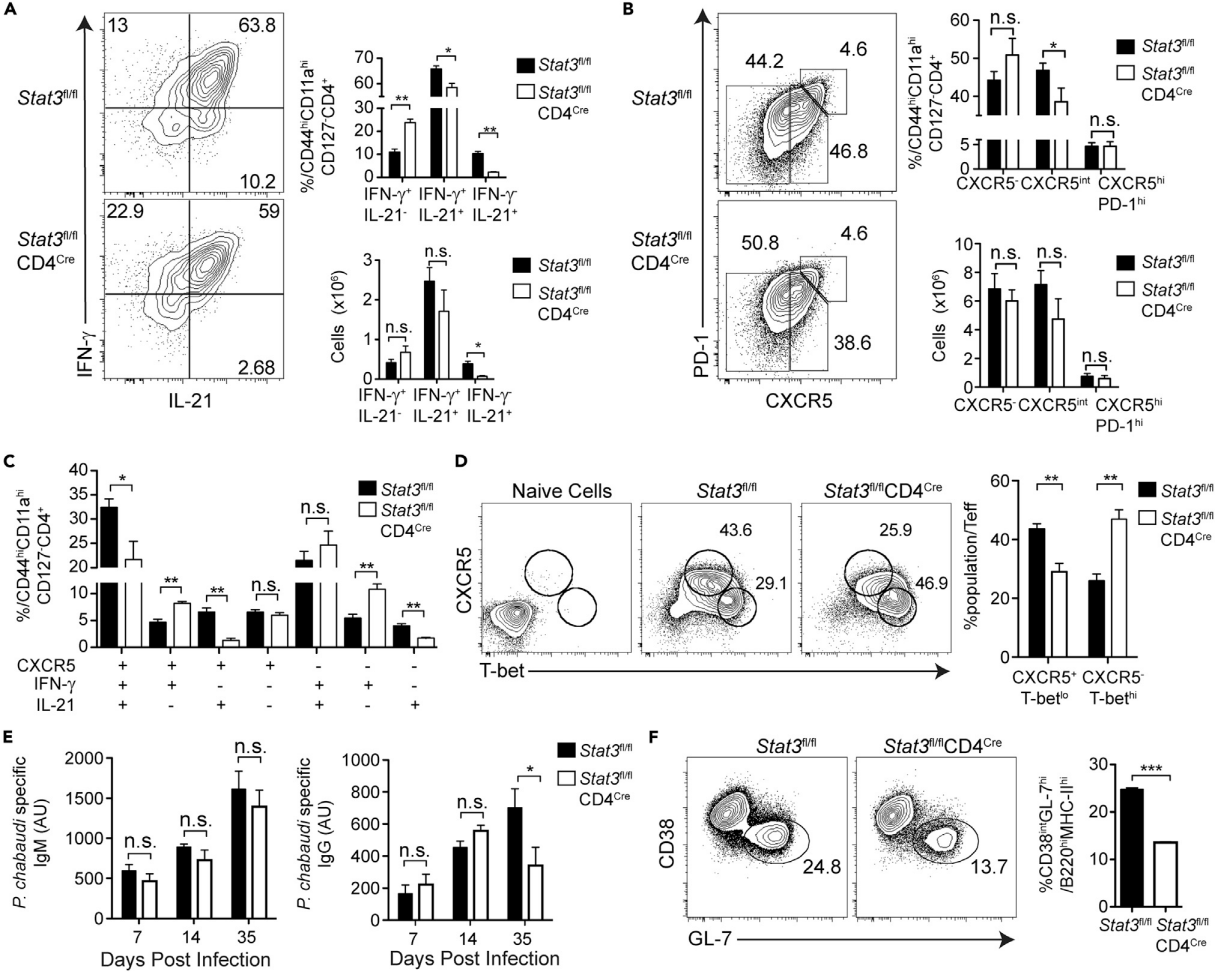
STAT3 Deficiency Reduces IFN-γ^+^IL-21^+^CXCR5^+^ Hybrid Teff and Increases Th1 Bias in *P. chabaudi* Infection (A and B) *Stat3*^fl/fl^CD4^Cre^ (TKO) and *Stat3*^fl/fl^ (WT) animals were infected, and splenocytes were analyzed at day 7 p.i. Expression of (A) IFN-γ and IL-21 and (B) PD-1 and CXCR5 in Teff. Bar graphs show percentages and numbers of subsets. (C) Boolean analysis of CXCR5^+^, IFN-γ^+^, and IL-21^+^ within WT (black bars) and STAT3 TKO (white bars) Teff. (D) Expression of CXCR5 and T-bet in Teff. Bar graph shows percentages Tfh-like (CXCR5^+^T-bet^lo^) and Th1-like (CXCR5^−^T-bet^hi^) subsets. (E) Bar graphs showing *P. chabaudi*-specific IgM (left) and IgG (right) from serum d35 p.i. (F) Expression of CD38 and GL-7 in B cells (B220^+^MHCII^+^) day 20 p.i. Bar graph shows numbers of GC B cells (CD38^lo^GL7^+^). Data representative of 3 experiments, 3–4 mice/group. Data are represented as mean ± SEM. ^∗^ p <0.05, ^∗∗^ p <0.01, ^∗∗∗^ p <0.001, ^∗∗∗∗^ p >0.05, n.s, not significant. See also Figures S5 and S6.

Because STAT3 promotes the Tfh phenotype (Batten et al., 2010; Choi et al., 2013; Nurieva et al., 2008; Ray et al., 2014), we hypothesized that STAT3 could also be a transcriptional regulator of the phenotype and/or function of hybrid Th1/Tfh cells. To test this hypothesis, we infected *Stat3*^fl/fl^CD4^Cre^ (STAT3 TKO) and *Stat3*^fl/fl^ (WT) animals with *P. chabaudi* or *P. yoelii* and analyzed splenocytes at day 7 or 10 p.i, respectively, by flow cytometry. STAT3 TKO mice infected with *P. chabaudi* showed a decrease in the percentage of IFN-γ^+^IL-21^+^ Teff, whereas the opposite was true for mice infected with *P. yoelii* (Figures 5A and S5A). We found no significant differences in the proportions of GC Tfh in either infection model (Figures 5B and S5B). However, using Boolean gating it became clear that STAT3 TKO mice infected with either *P. chabaudi* or *P. yoelii* both showed a reduction in the percentage of hybrid Th1/Tfh cells (IFN-γ^+^IL-21^+^CXCR5^+^, Figures 5C and S5C). There was a concomitant increase in the fraction of Th1-like cells (IFN-γ^+^IL-21^-^CXCR5^-^) in STAT3 TKO, and some small differences in the individual markers. In both infections, STAT3 TKO mice also had a significant reduction in IFN-γ^-^IL-21^+^ Teff, supporting reports that STAT3 signaling promotes IL-21 expression. Moreover, STAT3 TKO mice had an increase in the more Th1-like CXCR5^int/lo^T-bet^hi^ population compared with the two apparently separable populations seen in WT (Figures 5D and S5D). We interpret these data to suggest a continuum of plastic hybrid Th1/Tfh cells from a Th1-like to Tfh-like bias, rather than separate or terminally differentiated subsets. This would predict that cytokines that signal through STAT3 could shift the hybrid population over the course of infection. To test this, we blocked IL-6 and IL-27 signaling. Both cytokines signal through STAT3 and can influence Tfh and Th1 differentiation in other models (Batten et al., 2010; Sebina et al., 2017). Neutralization of IL-6 during infection of WT animals did not change the hybrid Th1/Tfh phenotype (Figure S6A). However, when T cells deficient in WSX-1 (IL-27Rα) were transferred into congenically marked recipients, which were then infected, the resulting divided Teff population (CD4^+^CTV^-^) contained more IFN-γ^+^IL-21^-^ Th1-like cells compared with WT donor cells (Figure S6B). This suggests that IL-27 is responsible for promoting IL-21 expression in IFN-γ^+^ T cells in *P. chabaudi* infection. IL-27Rα deficiency in T cells also strongly reduced GC Tfh but did not affect CXCR5^int^ T cells. Generation of *P. chabaudi*-specific antibody was also affected by STAT3 deficiency in T cells. IgG titers were significantly less at day 35 p.i. in STAT3 TKO mice, whereas the relative concentration of IgM was not affected (Figure 5E). In addition, the proportion of GC B cells was significantly reduced in STAT3 TKO mice at days 20 and 55 p.i. (Figure 5F, d55 not shown). *P. chabaudi*-infected STAT3 TKO mice had consistently prolonged parasitemia (Figure S6C) and pathology (Figure S6D). However, no STAT3 TKO mice died of *P. chabaudi* infection (n = 40). These results indicate that STAT3 regulates the phenotype and cytokine production of hybrid Th1/Tfh cells during *Plasmodium* infection through IL-27Rα signaling. However, STAT3 deficiency is detrimental for parasite control, prolonging pathology in the first infection.

### T-bet Expression Is Regulated by Bcl6, Blimp-1 and STAT3

**Figure 6:**
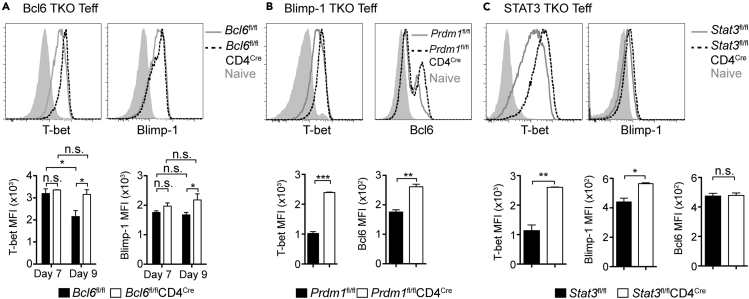
Bcl6, Blimp-1, and STAT3 Control T-bet Expression during *P. chabaudi* Infection TKO and WT animals were infected and splenocytes were analyzed. (A) Expression of T-bet (left) and Blimp-1 (right) in Teff from Bcl6 TKO (*Bcl6*^fl/fl^CD4^Cre^, dotted line), WT (*Bcl6*^fl/fl^, gray line), and naive (gray filled line) cells at day 9 p.i. Bar graphs show average MFI of T-bet and Blimp-1 at days 7 and 9 p.i. (B) Expression of T-bet (left) and Bcl6 (right) in Teff from Blimp-1 TKO (*Prdm1*^fl/fl^CD4^Cre^, dotted line) and WT (*Prdm1*^fl/fl^, gray line) animals and naive (gray filled line) cells at day 7 p.i. Bar graphs show average MFI of T-bet and Bcl6 at day 7 p.i. (C) Expression of T-bet (left) and Blimp-1 (right) expression in Teff from STAT3 TKO (*Stat3*^fl/fl^CD4^Cre^, dotted line) and WT (*Stat3*^fl/fl^, gray line) animals and naive (gray filled line) cells at day 7 p.i. Bar graphs show average MFI of T-bet, Blimp-1, and Bcl6 expression. Data representative of 3 experiments with 3–4 mice/group. Data are represented as mean ± SEM. ^∗^ p <0.05, ^∗∗^ p <0.01, ^∗∗∗^ p <0.001, ^∗∗∗∗^ p >0.05, n.s, not significant. See also Figure S7.

Based on the strong effect of T-bet deletion on IFN-γ production, we hypothesized that T-bet regulation could modulate the pathogenic potential of T cells *in vivo*. We previously showed that T-bet is downregulated from day 7 to day 9 p.i., even though day 9 is the peak of IFN-γ^+^ Teff numbers (Carpio et al., 2015). Therefore, we measured T-bet expression in all the TKO mice previously described. In Bcl6 TKO animals, T-bet expression was maintained at intermediate levels in Teff from day 7 to day 9 p.i., suggesting Bcl6 controls T-bet expression at the peak of infection (Figure 6A). Blimp-1 expression was also increased at day 9 p.i. in Bcl6 TKO. *Prdm1*^−/−^ Teff showed an increase in the expression of T-bet, as well as in Bcl6, at day 7 p.i. (Figure 6B). STAT3 TKO Teff also had more T-bet and Blimp-1 expression at day 7 p.i. (Figure 6C). Bcl6 expression was not affected in STAT3 TKO Teff in *P. chabaudi* infection; however, it was reduced on day 10 p.i. of *P. yoelii* infection of STAT3 TKO (Figure S5F). In conclusion, Bcl6, Blimp-1, and STAT3 work in concert to regulate the expression of T-bet, IFN-γ, CXCR5, and each other, in Teff during persistent *Plasmodium* infection.

As the hybrid Th1/Tfh phenotype is increased when infection lasts longer than 3 days, we investigated the functional phenotype of Teff in TKO mice during shorter infection. WT and TKO animals were infected, and one group of each was treated with MQ starting at day 3 p.i. (Figure S7). Data are quantified as a ratio of TKO over WT to illustrate the degree of the effect of removal of each transcription factor in the longer (NTx) or the shorter (+MQ) infection. Both Th1-like IFN-γ^+^IL-21^-^ and hybrid IFN-γ^+^IL-21^+^ Teff were significantly increased in the short-term infection in Bcl6 TKO mice compared with WT, showing a larger effect of Bcl6 on IFN-γ expression in shorter stimulation than longer (Figure S7A). Blimp-1 plays a larger role in longer infection, as only untreated infected Blimp-1 TKO (NTx), but not treated, had fewer IFN-γ^+^IL-21^-^ with a concomitant increase in hybrid IFN-γ^+^IL-21^+^, supporting its role in IL-10 expression. On the other hand, STAT3 regulates IFN-γ in both long and short infections. This is clearly shown in the strong increase of IFN-γ^+^IL-21^-^ Teff in STAT3 TKO mice compared with WT. Strikingly, GC Tfh generation was only affected by STAT3 deficiency in the shortened infection, supporting the previously described role of STAT3 in Tfh in acute infection (Figure S7B, Ray et al., 2014). Together, these results suggest that the role of each transcription factor is dependent on the duration of strong priming, presumably due to differential expression of cytokines and transcription factors driven by the milieu.

### Increasing Th1 Bias in Memory T Cells Correlates with Lower Parasitemia in Reinfection

**Figure 7:**
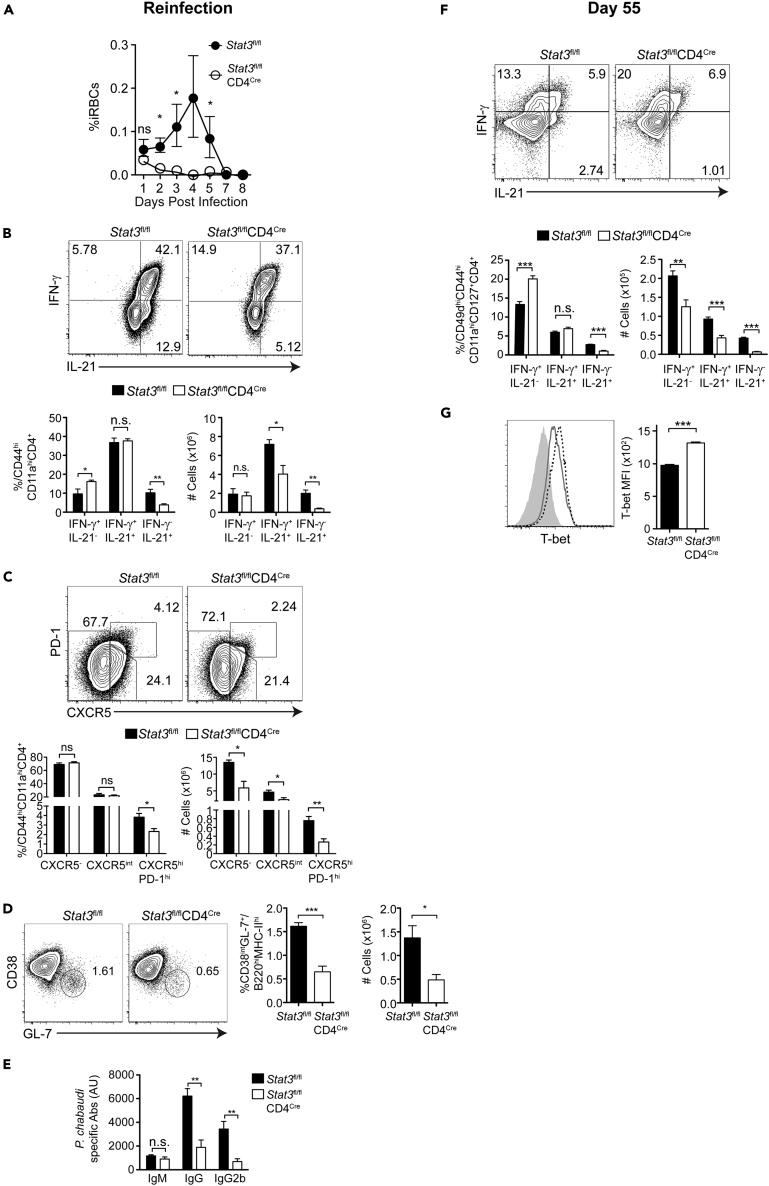
STAT3 TKO Mice Are Protected from Reinfection Despite Weaker Humoral Response STAT3 TKO (*Stat3*^fl/fl^CD4^Cre^) and WT (*Stat3*^fl/fl^) animals were infected. At day 60 p.i. both groups were treated with chloroquine (CQ) before reinfection at 6 weeks with 10^7^ iRBCs. (A) Parasitemia of WT (filled circles) and STAT3 TKO (open circles) animals. (B and C) Expression of (B) IFN-γ and IL-21 and (C) PD-1 and CXCR5 by antigen-experienced CD4 T cells (CD44^hi^CD11a^hi^) from STAT3 TKO and WT animals at day 7 post-re-infection. Bar graphs show percentages and numbers. (D) Expression of CD38 and GL-7 in B cells (B220^+^MHCII^+^) at day 7 post-re-infection. Bar graphs show percentages and numbers. (E) Bar graphs showing *P. chabaudi*-specific IgM, IgG, and IgG2b at day 7 post-re-infection. (F) Expression of IFN-γ and IL-21 in Tmem (CD44^hi^CD11a^hi^CD49d^hi^) from STAT3 TKO and WT animals at day 55 p.i. (first infection). Bar graphs show percentages and numbers. (G) Expression of T-bet in Tmem from WT (gray line) and STAT3 TKO (dotted black line) animals and naive (gray filled line) cells at day 55 p.i. Bar graph shows MFI of T-bet. Data representative of 2 experiments, 4–5 mice/group. Data in (A) are pooled from 2 independent experiments, 3–5 mice/group/experiment. Data are represented as mean ± SEM. ^∗^ p <0.05, ^∗∗^ p <0.01, ^∗∗∗^ p <0.001, ^∗∗∗∗^ p >0.05, n.s, not significant.

Th type-1 cytokines have a strong impact on parasitemia in mice and humans (Luty et al., 1999; Su and Stevenson, 2000), although less is known about re-infection. Given the increase of Th1 cells and effective clearance of persistent parasite in STAT3 TKO mice, we re-infected STAT3 TKO animals to test for immunity (Figure 7). To ensure parasite clearance after the first infection in both STAT3 TKO and WT, we treated with the anti-malarial drug chloroquine (CQ), which effectively eliminates low levels of *P. chabaudi* parasitemia (Hunt et al., 2004). STAT3 TKO mice controlled a high-dose second challenge (1 × 10^7^ iRBCs) completely, with infection becoming undetectable by day 3 post-reinfection (p.r.i, Figure 7A). WT mice showed significantly higher parasitemia that peaked around day 4 and was controlled by day 7 p.r.i. The proportion of IFN-γ^+^IL-21^-^ T cells was higher in STAT3 TKO mice at day 7 p.r.i, and the numbers of IFN-γ^+^IL-21^+^ Teff were less (Figure 7B). The numbers of both GC Tfh (Figure 7C) and GC B cells (Figure 7D) were significantly less in STAT3 TKO mice than WT at day 7 p.r.i. Importantly, the levels of *P. chabaudi*-specific IgG and Th1-driven isotype, IgG2b, were significantly less in STAT3 TKO mice than WT (Figure 7E). To determine if increased Th1 bias in the *stat3*^*−/−*^ Teff observed during the first infection was maintained into the memory phase, we analyzed antigen-experienced memory T cells (Tmem, CD11a^hi^CD49d^hi^CD44^hi^CD127^hi^) at day 55 p.i. Indeed, STAT3-deficient Tmem had higher percentages of IFN-γ^+^IL-21^-^ Th1-like cells (Figure 7F) and maintained higher expression of T-bet (Figure 7G) than WT.

**Figure 8:**
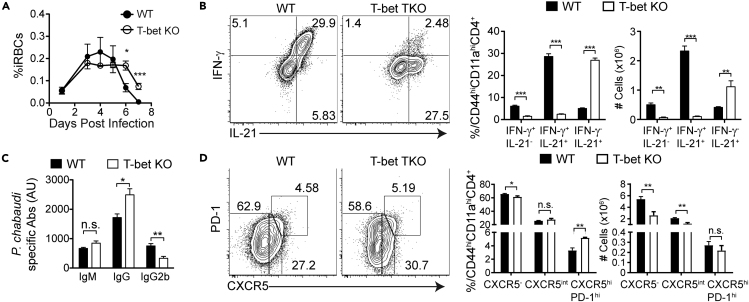
T-bet KO Mice Have Few Th1 Memory Cells, Reduced IgG2b, and Prolonged Parasitemia on Reinfection (A, B, and D) T-bet KO and WT animals were infected. At day 60 p.i. both groups were treated with chloroquine (CQ) before reinfection. After 3 weeks mice were re-infected with 10^7^ iRBCs. (A) Parasitemia of WT (filled circles) and T-bet KO (open circles) animals. Expression of (B) IFN-γ and IL-21 and (D) PD-1 and CXCR5 by antigen-experienced CD4 T cells (CD44^hi^CD11a^hi^) from T-bet KO and WT animals at day 7 post-reinfection. Bar graphs show percentages and numbers. (C) Bar graphs showing *P. chabaudi*-specific IgM, IgG, and IgG2b at day 7 post-re-infection. Data representative of 1 experiment, 3–5 mice/group. Data are represented as mean ± SEM. ^∗^ p <0.05, ^∗∗^ p <0.01, ^∗∗∗^ p <0.001, ^∗∗∗∗^ p >0.05, n.s, not significant.

The increase in Th1-like (IFN-γ^+^IL-21^-^) cells, decrease in *Plasmodium*-specific serum antibody, and concomitant very strong protection in STAT3 TKO mice support a role for Th1 cells rather than antibody in reinfection. Therefore, we tested the importance of Th1 cells in immunity by giving T-bet KO mice a second infection (Figure 8). T-bet KO mice showed prolonged parasite growth compared with WT mice, with days 6 and 7 p.r.i. remaining uncontrolled (Figure 8A). This was the opposite phenotype to STAT3 TKO, as predicted. Upon *P. chabaudi* reinfection, Teff in T-bet-deficient mice still produced IL-21, but little IFN-γ (Figure 8B). T-bet-deficient mice had increased levels of *P. chabaudi*-specific IgG, but lower levels of Th1-isotype IgG2b (Figure 8C). Furthermore, we observed a significant increase in the proportions of GC Tfh cells that could explain the aberrant isotype switching (Figure 8D). In summary, T cell-intrinsic STAT3 regulates the Th1 bias of memory T cells in *P. chabaudi* infection. Importantly, Th1 cells promote immunity, in addition to the role of pre-existing antibody, particularly IgG2b (Su and Stevenson, 2002).

## Discussion

Both Th1 and Tfh cells are required to eliminate parasites in *Plasmodium* infection. Previous work on the immune response to *P. chabaudi* shows that IFN-γ controls the height of the peak of parasitemia, whereas Tfh and IL-21 are required for antibodies to eliminate the parasite (Perez-Mazliah et al., 2015, 2017; Su and Stevenson, 2002; Gbedande et al., 2020; Meding and Langhorne, 1991). We have found that both types of effector functions are combined in one cell type in this infection (Carpio et al., 2015). Although there are certainly GC Tfh that make IFN-γ, we continue to term the multi-functional Teff cells found in persistent infections hybrid Th1/Tfh, rather than Th1-like Tfh, due to the larger effect and active regulation of T-bet (which controls their IFN-γ production) and the smaller effect of Bcl6 (suggesting a more Th1-like lineage), as well as their lack of the true GC Tfh (CXCR5^hi^PD-1^hi^) phenotype. It is important to note that in some staining combinations, two populations (i.e., CXCR5^int^T-bet^hi^, CXCR5^lo^T-bet^int^) appeared detectable within the hybrid population by fluorescence-activated cell sorting, as previously predicted by single cell RNA sequencing analysis (Lonnberg et al., 2017). While the populations are separable, as now clearly shown by CXCR6 staining of the Th1-like population (Soon MSF, et al, 2019), we would argue that the CXCR5^int^ population we detect here, and the two plastic populations within it, do not represent truly differentiated populations, but rather two ends of a continuum. However, we agree that this population can intuitively be termed more Th1- or Tfh-like, as has been the convention in the literature to date. The fact that each feature of these cells is actively regulated by so many transcription factors highlights their plasticity, as a necessity of adapting to the current infection-mediated cytokine milieu. Therefore, we conclude that the Teff population in this infection is not made up of subsets, but is a plastic, heterogeneous, hybrid population that is actively regulated by multiple inputs and transcription factors throughout the infection.

Protection from repeated episodes of malaria in humans correlates with serum IFN-γ and memory Th1 cells (Luty et al., 1999; Moormann et al., 2013; Stephens and Langhorne, 2010). CD4 T cells in adults from malaria-endemic areas also express cytokines of multiple lineages including IFN-γ, IL-10, and IL-21, even in cells with a Tfh-like phenotype (Obeng-Adjei et al., 2015; Roetynck et al., 2013). Human Teff expressing both CXCR3 and CXCR5 and mouse CXCR5^+^ Teff expressing markers of a high level of activation (Ly6C, NK1.1) can help B cells make antibody; however, they are less effective helper cells *in vitro* than those expressing only CXCR5 (Obeng-Adjei et al., 2015; Zander et al., 2017; Wikenheiser et al., 2018). In humans, CXCR3^+^CXCR5^+^ T cells were not shown to correlate with *Plasmodium*-specific antibody levels (Obeng-Adjei et al., 2015). Strikingly, hybrid Th1/Tfh cells (ICOS^+^CXCR3^+^CXCR5^+^) did correlate with antibody levels in influenza, where they were also shown to contain IFN-γ^+^IL-21^+^ T cells (Bentebibel et al., 2013). Acute infections, like those caused by *Listeria*, can induce a stable Th1 memory phenotype (Curtis et al., 2010; Hale et al., 2013), whereas chronic LCMV and tuberculosis infections have T cell responses skewed away from a committed Th1 phenotype, and toward concomitant expression of Tfh markers in mice (Crawford et al., 2014; Li et al., 2016). Therefore, Th phenotype plasticity appears to be a shared feature of the immune response to persistent infections and has been shown to be beneficial in control of tuberculosis (Khader et al., 2007; O'Shea and Paul, 2010). Similarly, our data suggest that preserving plasticity would be optimal for protection.

We found that T-bet regulates cytokine production in T cells in *P. chabaudi* infection. In addition, the expression of T-bet is highly regulated, including by STAT3, Blimp-1, and Bcl6, presumably to avoid immunopathology. T-bet expression kinetics also support ongoing regulation. We previously observed that T-bet is downregulated before day 9, the peak of *Ifng*^+^ T cell expansion (Carpio et al., 2015). Here, we show that this downregulation occurs in a Bcl6-dependent manner. Although T-bet is generally induced by STAT1 in CD4 T cells (Afkarian et al., 2002), and upregulated upon IL-12 signaling through STAT4, neither STAT4 nor STAT1 deficiency negatively regulated the hybrid cytokine profile in T cells. This observation suggests that T-bet in this infection is induced primarily by TCR signaling and IL-18 and/or that there is some redundancy between STATs. T-bet regulation is a critical focus in the control of Th plasticity in this persistent infection.

The hybrid T cell dominant at the peak of infection facilitates strong early cellular and humoral responses. However, the two types of responses clearly also regulate one another. T-bet in T cells has recently been shown to impair GC Tfh cell differentiation and GC formation (Ryg-Cornejo et al., 2016), though it is critical in B cells (Ly et al., 2019). On the other hand, a recent study concluded that T-bet and STAT4 are actually required for GC Tfh development and GC formation during acute viral infection (Weinstein et al., 2018). We confirm that STAT4 is also required for GC Tfh in *P. chabaudi* infection. We did not detect any change in the number of GC Tfh in T-bet-deficient animals infected with *P. chabaudi* during the primary infection as seen in *Plasmodium berghei* ANKA. However, we observed a significant reduction in the percentage of GC Tfh in the secondary infection in T-bet KO mice. T-bet, presumably in its capacity for driving IFN-γ in T cells and thereby promoting production of the IgG2 isotype of antibody, was essential for full parasite control in both the first and second infections. Clearly, the regulation of T-bet and IFN-γ is a high priority for promoting an effective Teff response and survival of the animals given the multiple transcription factors involved. However, the production of different cytokines during continued infection leads to functional T cell plasticity due to the unique regulation of functional attribute, and overlap in TCR, co-stimulation, and cytokine signaling cascades.

Overlapping signaling cascades control the balance of Th1 and Tfh programs (Weinmann, 2014). IL-12, the primary cytokine responsible for induction of Th1 cells can be essential for generation of Tfh *in vivo* and can also induce IL-10 (Saraiva et al., 2009; Weinstein et al., 2018). In addition, *in vitro*-generated Th1 cells transiently express Bcl6 and IL-21, whereas Tfh transiently express T-bet (Fang et al., 2018; Nakayamada et al., 2011). T-bet can bind and inhibit the Tfh-driving transcription factor, Bcl6 (Oestreich et al., 2012). On the other hand, T cells that express T-bet will not necessarily express IFN-γ, particularly if they also express the transcriptional Bcl6 (Oestreich et al., 2011). Although we previously showed that Bcl6 levels correlate with the level of *Ifng* transcription in intact mice (Carpio et al., 2015), the Bcl6 TKO Teff do not have more IFN-γ protein by intracellular cytokine staining here. Both STAT3 and Bcl6 are reported to be required for Tfh differentiation, whereas Blimp-1 inhibits both Th1 and Tfh differentiation (Cimmino et al., 2008; Johnston et al., 2009; Ma et al., 2012; Nurieva et al., 2009; Ray et al., 2014). However, in our studies, deficiency in either Bcl6 or STAT4 in T cells eliminated GC Tfh, whereas STAT3 deficiency did not. STAT3 deficiency did, however, reduce the proportions of GC Tfh in the setting of a shorter *P. chabaudi* infection, in agreement with previous reports that used acute infections as stimuli (Ray et al., 2014). In addition, we consistently observed that STAT3-deficient T cells did not develop into Tfh-like IFN-γ^-^IL-21^+^ Teff in both *P. yoelii* and *P. chabaudi* infections. The shorter infection also resulted in an increase of GC B cells, similar to the increase in GC B cells induced by inactivated *P. berghei* ANKA (Ryg-Cornejo et al., 2016). Given the differences in regulation in shorter versus longer infections shown here, the regulation of *prdm1* expression by type1-I IFN is likely central to the regulation of terminal Tfh differentiation and the increased plasticity during prolonged infection (Zander et al., 2016). Bcl6 also reduced the level of expression of CXCR5, whereas Blimp-1 had the opposite effect. Our data suggest that Bcl6 and Blimp-1 have a stronger effect on regulation of CXCR5 expression than on production of IFN-γ or IL-21. This may be explained by the inhibition by Bcl6 of microRNAs that control *cxcr5* expression (Yu et al., 2009).

The most compelling result here is that skewing the hybrid-lineage cells toward a more committed Th1 phenotype in STAT3 TKO dramatically sped up clearance of parasitemia on reinfection. Supporting this interpretation, T-bet-deficient mice, with less Th1 bias and more IFN-γ^-^IL-21^+^ T cells, were significantly slower at clearing a second infection. While the protection did not correlate with antibody levels, we did not rule out a role for antibody, particularly affinity maturation and isotype switching, in the improved immunity of STAT3 TKO. The mechanism of evolutionary pressure regulating this balance becomes clear in that the shift toward Th1 in STAT3 TKO animals, as well as the shift away from Th1 in the T-bet KO, as also shown for *P. berghei* ANKA, both prolong high parasitemia and pathology in the first infection. STAT3 has been previously suggested to be able to promote Tfh and inhibit Th1 differentiation (Ray et al., 2014; Wu et al., 2015). We found that IL-6 had no impact on the differentiation of Teff. Although IL-27Rα-deficient animals have long been known to have a hyperactive T cell response to pathogens including *Plasmodium*, we have shown that IL-27Rα on T cells regulates the balance between IFN-γ and IL-21 and GC Tfh differentiation, which is supported by the work of others (Batten et al., 2010; Guthmiller et al., 2017; Gwyer Findlay et al., 2014; Hibbert et al., 2003; Kane et al., 2014; Ma et al., 2012; Stumhofer et al., 2007). Supporting our analysis, IL-27 has been shown to be made by CD4 T cells in *Plasmodium* infection (Kimura et al., 2016). It would be of great interest to dissect the molecular pathway that IL-27 uses to guide cytokine production versus its effect on GC Tfh differentiation, for example, the relative role of STAT1 and STAT3 in each. Recent studies have shown that IL-12 can promote STAT3 association with the *bcl6* and *il21* loci in T cells *in vitro*, suggesting a possible signaling pathway for the regulation of plastic Th1 and Tfh populations in the context of complex cytokine milieus (Powell et al., 2019).

Previous studies have demonstrated that the delicate balance of a *P. chabaudi*-infected animal's life or death is regulated by CD4 T cells, as is the case in other persistent infections such as tuberculosis and toxoplasmosis (Caruso et al., 1999; Denkers and Gazzinelli, 1998; Stephens et al., 2005). Animals deficient in either of the pro-Th1 factors IL-12 or IFN-γ, or the regulatory cytokine IL-10, are more susceptible to die of *P. chabaudi* infection, even though it is a normally mild infection in mice (Li et al., 1999; Su and Stevenson, 2000, 2002). T-bet has been shown to be required for control of *P. berghei* ANKA parasitemia but is also essential for pathogenesis of experimental cerebral malaria (Oakley et al., 2013). Although we did not see any mortality from an increase in Th1-type cells in the infected STAT3 TKO, mice that either had more (STAT3) or less (T-bet) IFN-γ^+^IL-21^-^ Th1-type cells had prolonged pathology. Therefore, although our data, and the human literature, suggest that a stronger Th1 response is beneficial for immunity to *P. chabaudi*, it remains to be tested if the combination of Th1/Tfh and regulatory cytokines into one cell type represents an evolutionary benefit, particularly in the first infection, which is likely to drive evolution the most (next to pregnancy malaria). Further work considering the finely-tuned balance required to ensure host survival is needed to determine if this hybrid response is maladaptive.

In summary, persistent *Plasmodium* infection drives generation of a plastic mixed-lineage T cell with characteristics of both uncommitted Th1 (T-bet^int^) and pre-Tfh (CXCR5^int^), that is balanced by STAT3, Bcl6, Blimp-1, and T-bet, which coordinate the relative degree of antibody and IFN-γ responses for optimal pathogen control and host survival. Changing this balance toward Th1 in the first infection may prolong pathology, whereas it promotes sterilizing immunity in the longer term, suggesting a potential direction for vaccine development.

### Limitations of the Study

Defining Th1 and Tfh cells by the markers they express can both enhance and limit our understanding. However, we have focused on three markers (CXCR5, IFN-γ, and IL-21) with functional consequences and assays with good discrimination of positive and negative. In addition, most of the Teff population appears to express both T-bet and Bcl6; however, flow cytometry does not report on transcriptional activation. As both Th1/Tfh and GC Tfh express both CXCR5 and CXCR3, it will be important to study the location of each cell type and interactions with B cells *in vivo* in our next study. Although we have used multiple models of rodent malaria, and the predictions from these models are often predictive of human malaria immunology (Stephens et al., 2012; Gbedande et al., 2020), there are likely to be differences of degree in *P. falciparum* infection. These models have the potential to inform other immune environments including other persistent pathogens and the response to transformed cells *in vivo*.

### Resource Availability

#### Lead Contact

Further information and requests for resources and reagents should be directed to, and will be fulfilled by, the Lead Contact, Robin Stephens (rostephe@utmb.edu).

#### Materials Availability

This study did not generate new unique reagents.

#### Data and Code Availability

This study did not generate/analyze datasets/code.

## Methods

All methods can be found in the accompanying Transparent Methods supplemental file.

## Supplemental Information

Document S1. Transparent Methods and Figures S1–S7
