# Supplementary material for: T Helper Plasticity Is Orchestrated by STAT3, Bcl6, and Blimp-1 Balancing Pathology and Protection in Malaria
Source: iScience. 2020 Jun 24;23(7):101310. doi: 10.1016/j.isci.2020.101310 (PMC7339051; doi:10.1016/j.isci.2020.101310)
Supplement: Document S1. Transparent Methods and Figures S1–S7 [file mmc1.pdf]

iScience, Volume *23*

Supplemental Information

T Helper Plasticity Is Orchestrated by STAT3, Bcl6, and Blimp-1 Balancing Pathology and Protection in Malaria

Victor H. Carpio, Florentin Aussenac, Lucinda Puebla-Clark, Kyle D. Wilson, Alejandro V. Villarino, Alexander L. Dent, and Robin Stephens

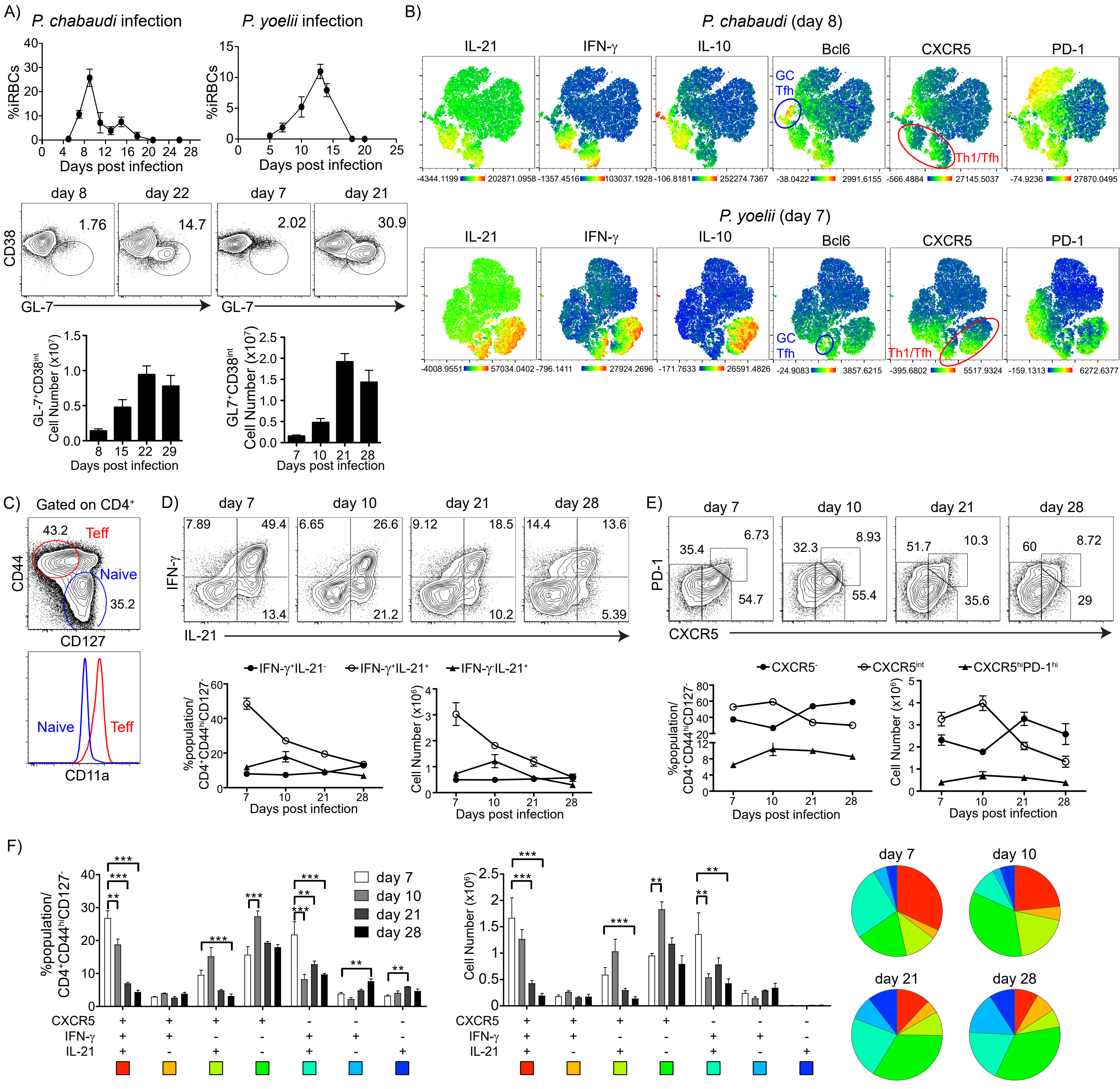

Figure S1. T **helper differentiation during** *P. chabaudi* **and** *P. yoelii* **infections.** *Related to Figure 1.* C57BL/6J mice were infected with either *P. chabaudi* or *P. yoelii* 17XNL and splenocytes analyzed on days indicated. (**A**) Parasitemia, and expression of CD38 and GL-7 in B cells (B220$^+$MHCII$^+$). Bar graph shows numbers of GC B cells (CD38^{lo}GL7$^+$) at indicated days. (**B**) Density t-SNE plots of CD4$^+$ T cells from C57BL/6J mice infected with *P. chabaudi* at day 8 p.i. or *P. yoelii* at day 7 p.i. Plots show 10^5 representative T cells from each of 3 mice, concatenated and overlaid with the expression of selected markers. (**C**) Expression of CD44, CD127, and CD11a in CD4$^+$ T cells at day 8 of *P. chabaudi* infection showing concordance of CD127$^-$ and CD11a^{hi} as markers of activation. Expression of (**D**) IFN-γ and IL-21 or (**E**) PD-1 and CXCR5 in Teff during *P. yoelii* infection. Line graphs show percentage (left) and numbers (right) of subsets over time. (**F**) Boolean gating of CXCR5$^+$, IFN-γ$^+$, and IL-21$^+$ of Teff in *P. yoelii* infection at each time point. Pie charts show the distribution of subsets on each day. Bar graphs show the percentages and cell numbers of the subsets on each day. Data representative of 2 experiments with 3 mice/group. Data are represented as mean ± SEM.

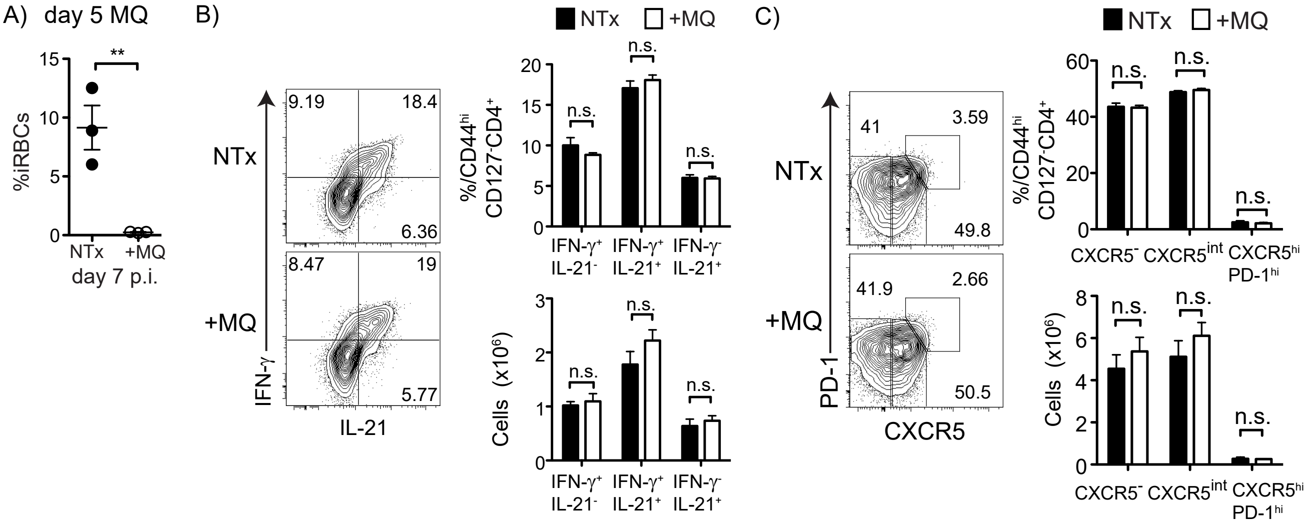

Figure S2. Stopping the infection on day 5 post-infection has no effect on hybrid Th1/Tfh cell phenotype. *Related to Figure 2*. C57BL/6J mice were infected, and one group was treated with mefloquine (MQ) daily starting day 5, and splenocytes were analyzed at day 7 p.i. (**A**) Parasitemia on day 7 p.i. from untreated (NTx, black filled circles) and treated (+MQ, open circles) groups. Expression of (**B**) IFN-γ and IL-21, or (**C**) PD-1 and CXCR5 in Teff. Bar graphs show percentage of Teff (top) and numbers (bottom). Data representative of 2 experiments with 3 mice/group. Data are represented as mean ± SEM.

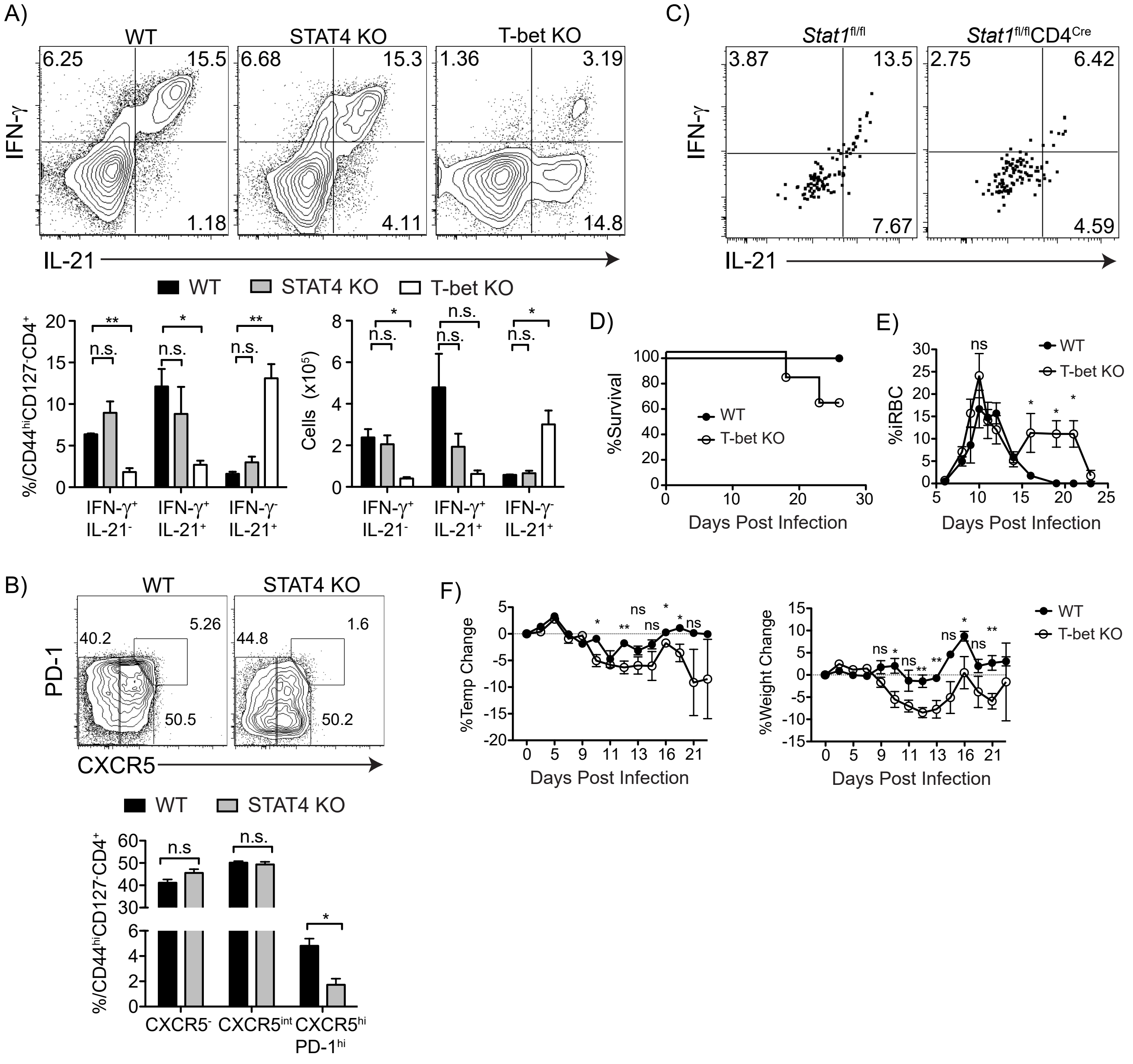

Figure S3. T-bet, but not STAT4 nor STAT1, is required for IFN-γ production by hybrid Th1/Tfh. *Related to Figure 3*. (**A**) C57BL/6J (WT), STAT4 KO, and *Tbx21* (T-bet) KO mice were infected and splenocytes were analyzed at day 7 p.i. Contour plots and bar graphs show expression of IFN-γ and IL-21 in Teff from WT (black bar), STAT4 KO (gray bar) and T-bet KO (white bar). (**B**) Expression of PD-1 and CXCR5 in Teff from WT and STAT4 KO mice at day 7 p.i. Below, bar graphs show percentages. (**C**) Splenocytes from uninfected *Stat1*$^{fl/fl}$CD4^{Cre} (STAT1 TKO) or *Stat1*$^{fl/fl}$ (WT) were labeled with cell trace violet (CTV) and adoptively transferred into Ly5.1 (CD45.1) congenic mice, which were then infected with *P. chabaudi*. Expression of IFN-γ and IL-21 in divided Teff (CTV$^-$ gated) on day 8 p.i. (**D**) Survival curve and (**E**) Parasitemia of WT (filled circles) and T-bet KO (open circles) groups. (**F**) Temperature and weight loss of infected WT and T-bet KO groups. Data representative of 2 experiments with 3-8 mice/group for (**A**, **D**, **E**, and **F**) and 1 experiment with 4-5 mice/group for (**C**). Data are represented as mean ± SEM.

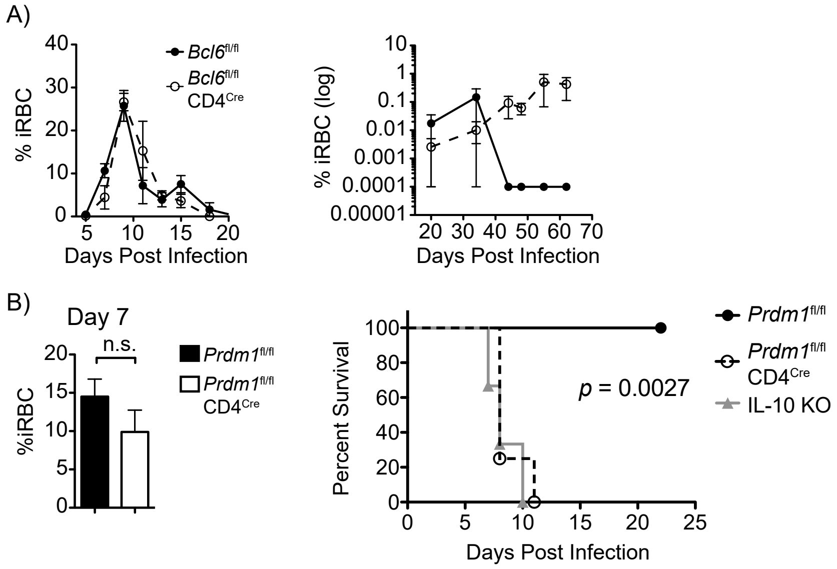

Figure S4. Roles of Bcl6 and Blimp in T cell differentiation during *P. chabaudi* infection. *Related to Figure 4***.** (**A**) *Bcl6*$^{fl/fl}$CD4^{Cre} (TKO, open circles) and *Bcl6*$^{fl/fl}$ (WT, filled circles) animals were infected and parasitemia was measured for 2 months. (**B**) *Prdm1*$^{fl/fl}$CD4^{Cre} (Blimp-1 TKO) and *Prdm1*$^{fl/fl}$ (WT) animals were infected and parasitemia was measured at day 7 p.i. Survival of WT (filled circles), Blimp-1 TKO (open circles) and IL-10 KO (gray triangles). Data representative of 3 experiments, 3-4 mice/group. Data are represented as mean ± SEM.

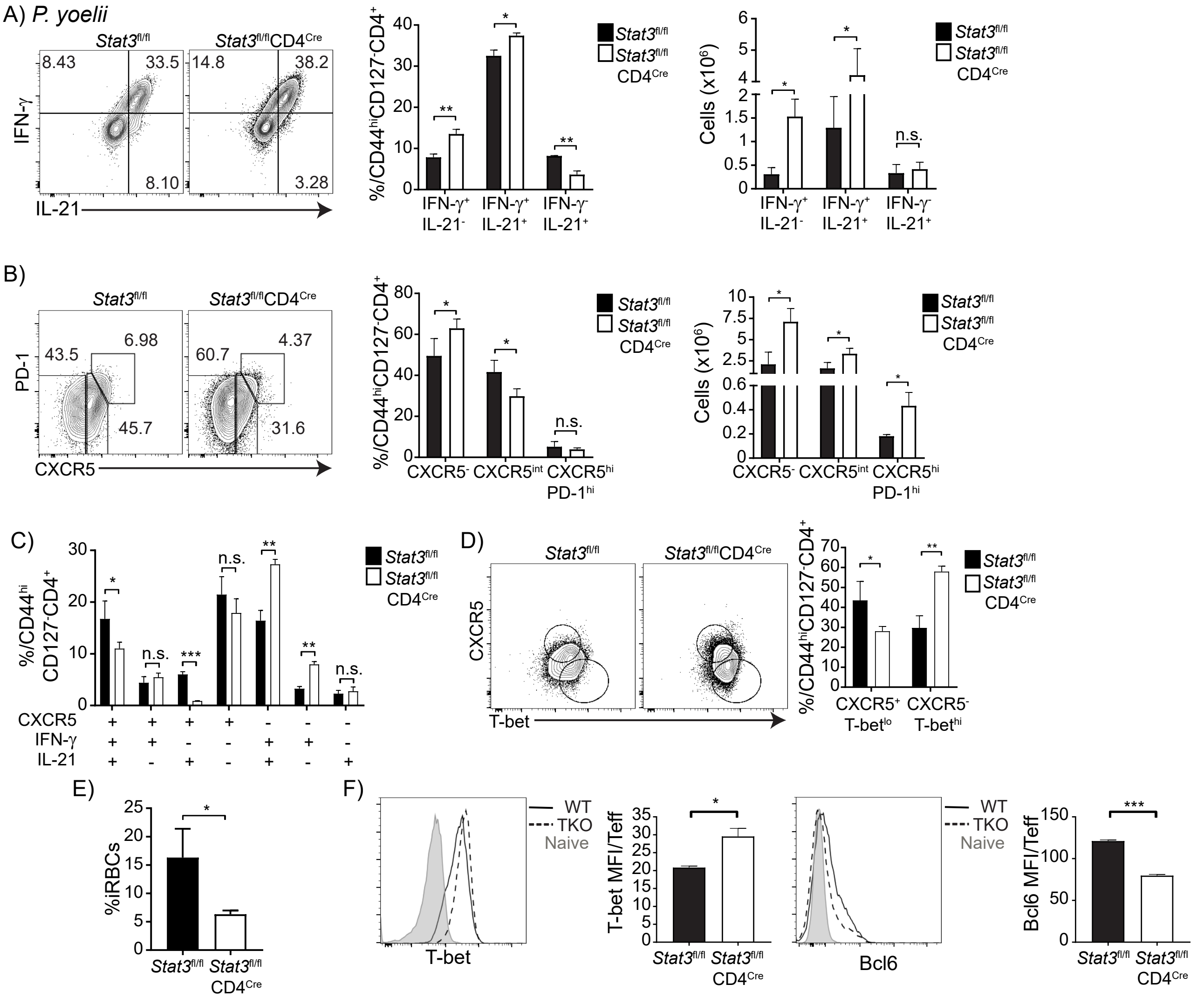

Figure S5. ***P. yoelii*****-infected STAT3 TKO mice show similar T cell phenotypes to** ***P. chabaudi*** **infection.** *Related to Figure 5*. *Stat3*$^{fl/fl}$CD4^{Cre} (TKO) and *Stat3*$^{fl/fl}$ (WT) animals were infected with *P. yoelii* 17XNL and splenocytes were analyzed at day 10 p.i. Expression of (**A**) IFN-γ and IL-21, or (**B**) PD-1 and CXCR5 gated on Teff. Bar graphs show percentages and numbers per spleen. (**C**) Boolean gating of CXCR5^{+}, IFN-γ^{+}, and IL-21^{+} within WT (black bars) and STAT3 TKO (white bars) Teff. (**D**) Contour plots show expression of CXCR5 and T-bet in Teff. Bar graph shows percentages of Tfh-like (CXCR5^{+}T-bet^{lo}) and Th1-like (CXCR5^{-}T-bet^{hi}) Teff. (**E**) Parasitemia of WT (black bars) and STAT3 TKO (white bars) animals on day 10 p.i. (**F**) Histograms showing T-bet (left) and Bcl6 (right) expression in Teff from STAT3 TKO (dotted line) and WT (black line) animals, and naive (gray filled line) cells. Bar graphs shows average MFI of T-bet and Bcl6. Data representative of 1 experiment with 2-3 mice/group. Data are represented as mean ± SEM.

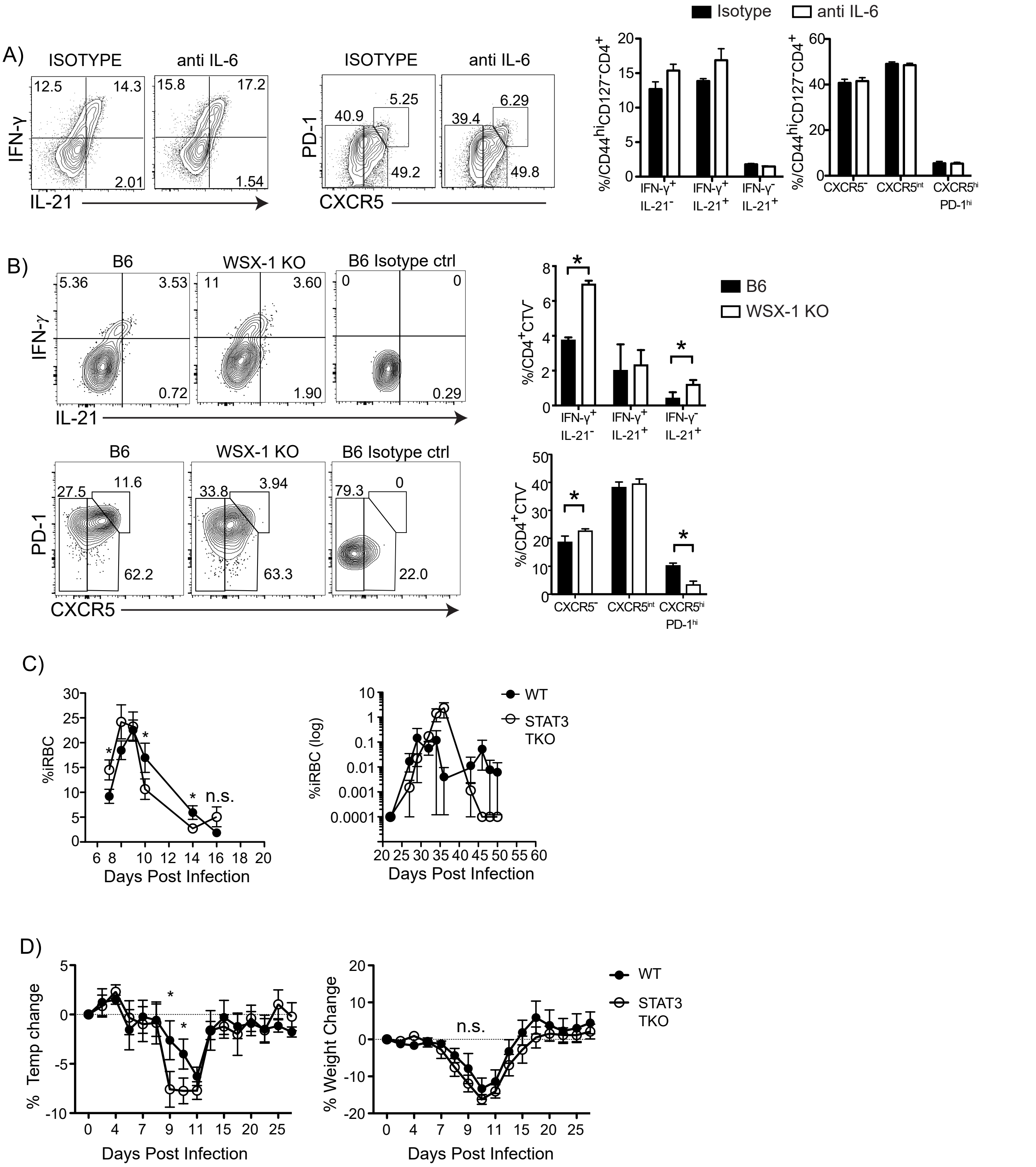

Figure S6. WSX-1 deficiency increases Th1-like Teff. *Related to Figure 5*. (**A**) C57BL/6J mice (n=5/group) were infected with *P. chabaudi* and treated with anti-IL-6 or isotype control antibody. Expression of IFN-γ and IL-21, or PD-1 and CXCR5 in Teff at day 7 p.i. in splenocytes from treated animals (**B**) Splenocytes from uninfected WSX-1 KO or C57BL/6J were labeled with cell trace violet (CTV) and adoptively transferred into Thy1.1 congenic mice, which were then infected with *P. chabaudi*. Expression of IFN-γ/IL-21 and PD-1/CXCR5 in CTV^{-} gated Teff on day 8 p.i. (**C**, **D**) *Stat3*$^{fl/fl}$CD4^{Cre} (TKO) and *Stat3*$^{fl/fl}$ (WT) animals were infected. (**C**) Parasitemia of WT (filled dots) and STAT3 TKO (open circles) animals. (**D**) Temperature and weight loss of WT and STAT3 TKO animals are shown as percentages of starting value. (**A**, **B**) Data representative of 2 experiments, 3-5 mice/group. (**C**, **D**) Data representative of 3 experiments, 3-8 mice/group. Data are represented as mean ± SEM.

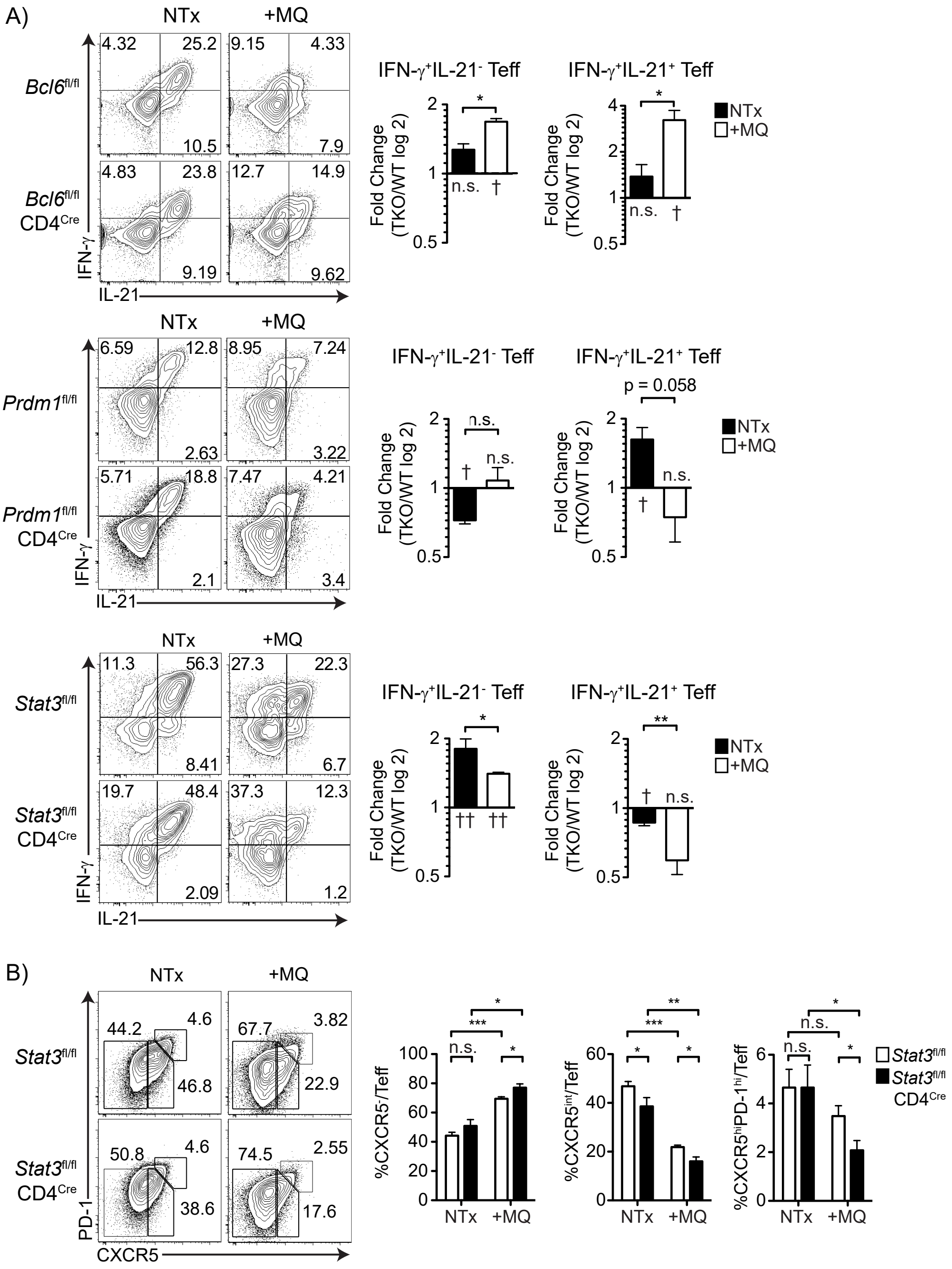

Figure S7. T cell response to long or shortened *P. chabaudi* infection in Bcl6, Blimp-1, and STAT3 TKO mice. *Related to Figure 6*. TKO and WT animals were infected and given mefloquine (+MQ) starting on day 3 p.i. or left untreated (NTx). Splenocytes were harvested and analyzed by flow cytometry at day 7 p.i. (**A**) Contour plots show expression of IFN-γ and IL-21 in Teff. Bar graphs show difference of IFN-γ^+IL-21^- and IFN-γ^+IL-21^+ Teff (average of fold change difference between TKO and WT (log 2 of %TKO/%WT)) from NTx (black bars) or +MQ (white bars) from Bcl6 TKO (top), Blimp-1 TKO (middle), or STAT3 TKO (bottom). (Intracellular cytokine staining in STAT3 TKO was the only one done with commercially prepared secretion inhibitor, hence higher cytokine staining) (**B**) Expression of PD-1 and CXCR5 in Teff from STAT3 TKO and WT mice. Bar graphs show percentages from STAT3 TKO (black bars) or WT (white bars). Data representative of 2 experiments with 3 mice/group. † $p < 0.05$, ††$p < 0.01$ are statistical significance of the difference between WT and TKO mice for each group. Data are represented as mean ± SEM.

Supplemental Information

Transparent Methods

Experimental Model and Subject Details

C57BL/6J (B6), B6.129S1-*Stat3*^{tm1Xyfu}/J (STAT3$^{fl/fl}$), B6.129-*Prdm1*^{tm1Clme}*/J* (Blimp-1$^{fl/fl}$), and B6.129S6-*Tbx21*^{tm1Glm}/J (T-bet KO) mice were purchased from The Jackson Laboratory (Bar Harbor, ME) and bred to B6.Cg-Tg (CD4-Cre)1Cwi N9 mice from Taconic (Hudson, NY). Bcl6$^{fl/fl}$ x CD4-Cre mice (Indiana University School of Medicine, Indianapolis, IN) were bred at UTMB. Six to twelve-week-old animals of both sexes were used for all experiments. All mice were maintained in our specific pathogen free animal facility with *ad libitum* access to food and water. All animal experiments were carried out in compliance with the protocol specifically approved for this study by the University of Texas Medical Branch Institutional Animal Care and Use Committee. Mice were infected i.p. with 10^5 (or 10^7 for re-infection) *P. chabaudi chabaudi* (AS; courtesy of Jean Langhorne (Francis Crick Institute, London, UK)) or 10^5 *P. yoelii* (clone 17XNL; MR4/ATCC) infected red blood cells (iRBCs). Parasites were counted in thin blood smears stained with Giemsa (Sigma, St. Louis, MO) by light microscopy. In some experiments, mice were treated with mefloquine hydrochloride (MQ, 4mg/kg body weight, Sigma, St. Louis, MO) by oral gavage daily five times or until the mice were euthanized. In some experiments (STAT3 TKO) mice were treated with 50 mg/kg body weight per animal of Chloroquine (CQ) in saline (both from Sigma) every other day for a total of three times, starting 10 weeks p.i.

Flow Cytometry and Adoptive Transfer

Single-cell suspensions from spleens were made in Hank's Balanced Salt Solution (Gibco, Life Technologies, Grand Island, NY), with added HEPES (Sigma), followed by red blood cell lysis buffer (eBioscience, San Diego, CA). Multicolor panels including anti-CXCR5 were stained in

PBS + 0.5% BSA + 0.1% sodium azide + 2% Normal Mouse Serum (NMS) and 2% FBS (Sigma, St. Louis, MO). Rat anti-mouse purified CXCR5 (2G8, BDbioscience, San Jose, CA, 1 hr., 4°C) was followed by biotin-conjugated AffiniPure Goat anti-rat (H+L, Jackson ImmunoResearch, West Grove, PA, 30 min, 4°C) followed by Streptavidin-eFluor 450, –PE or –Brilliant Violet 650 (BV650). As described in Crotty et al., the third step included the other antibodies (Crotty, 2014). Combinations of FITC–, phycoerythrin (PE)–, Peridinin Chlorophyll Protein Complex (PerCP)-Cyanine (Cy)5.5, PE/ Cyanine 7 (Cy7), Allophycocyanin (APC) monoclonal antibodies (all from eBioscience, San Diego, CA), and CD127-PE/Cy5, CD44-Brilliant Violet 785 (Biolegend, San Diego, CA) were used. For B cell staining we used B220-PE/Cy5, MHC-II (I-A/I-E)-APC, CD38-PE, GL-7-FITC (all from eBioscience, San Diego, CA). For intracellular staining, total cells were stimulated for 2 h with phorbol myristate acetate (PMA, 50 ng/mL), Ionomycin (500 ng/mL), and Brefeldin A (10 μg/mL, all from Sigma) in complete Iscove's Media 10% FBS, 2mM L-glutamine, 0.5 mM sodium pyruvate, 100 U/ml penicillin, 100ug/ml streptomycin, 50 μM 2-β-Mercaptoethanol (all from Gibco, LifeTechnologies). Figures 4B and S7A STAT3 TKO used GolgiPlug (BDbioscience) in place of Brefeldin A solution. Cells were fixed in 2% paraformaldehyde (Sigma), permeabilized using Permeabilization buffer (Perm buffer, eBioscience) and incubated for 40 minutes with anti-IFN-γ-Brilliant Violet 605 (XMG1.2), T-bet-eFluor 660 or -PerCP-Cy5.5 (eBio4B10, eBioscience), Bcl6-Alexa Fluor 488 or –PE (K112-91), and/or Blimp-1-Alexa Fluor 647 (6D3, BDbioscience). For IL-21 staining, cells were incubated with recombinant mouse IL-21R-Fc chimera (1 μg, 40 min., R&D systems, Minneapolis, MN in Perm buffer), washed twice in Perm buffer followed by AlexaFluor647 goat anti-human IgG $F(ab')_2$ (0.3 μg, 30 min, Jackson ImmunoResearch, West Grove, PA) in Perm buffer. After three washes in Perm buffer, cells were resuspended in FACS buffer and collected on a LSRII Fortessa

at the UTMB Flow Cytometry and analyzed in FlowJo versions 9.4.11, 10.5.3 (TreeStar, Ashland, OR). Compensation was performed in FlowJo using single CD4 stained splenocytes. Cell Trace Violet (CTV, Invitrogen) staining of splenocytes was done in calcium- and magnesium-free PBS at 10^7 cells/ml with 5μM CTV for 10 minutes at 37°C in the dark with periodic shaking, then quenched with Fetal Calf Serum. After washing, 2 x10^6 cells were transferred into each mouse i.p. Reagents are listed in the next table.

REAGENT or RESOURCE	SOURCE	IDENTIFIER
Antibodies		
Purified Rat Anti-Mouse CXCR5 (Clone 2G8)	BD Bioscience	Cat No. 551961, RRID:AB_394302
Biotin-SP (long spacer) AffiniPure Goat Anti-Rat IgG (H+L)	Jackson ImmunoResearch Labs	Cat No. 112-065-167, RRID:AB_2338179
eBioscience™ Streptavidin eFluor™ 450 Conjugate	Thermo Fisher Scientific	Cat No. 48-4317-82, RRID:AB_10359737
PE anti-Streptavidin	Biolegend	Cat No. 410503, RRID:AB_2571914
Brilliant Violet 650™ Streptavidin	Biolegend	Cat No. 405232
PE/Cy7 anti-moue CD279 (PD-1)	Biolegend	Cat No. 109110, RRID:AB_572017
GL-7 Monoclonal Antibody (GL-7), Alexa Fluor 488, eBioscience™	Thermo Fisher Scientific	Cat No. 53-5902-82, RRID:AB_2016717
CD38 (clone HB7), PE, eBioscience™	Thermo Fisher Scientific	Cat No. 12-0388-42, RRID:AB_1518748
CD11a (LFA-1alpha) (clone HI111), FITC, eBioscience™	Thermo Fisher Scientific	Cat No. 11-0119-42, RRID: AB_10596521
PE/Cy5 anti-mouse CD127 (IL-7Rα)	Biolegend	Cat No. 135016, RRID:AB_1937261
Brilliant Violet 785™ anti-mouse/human CD44	Biolegend	Cat No. 103059, RRID:AB_2571953
CD45R (B220) (Clone RA3-6B2), PE-Cyanine5, eBioscience™	Thermo Fisher Scientific	Cat No. 15-0452-82, RRID:AB_468755
MHC Class II (I-A/I-E) (clone M5/114.15.2), APC, eBioscience™	Thermo Fisher Scientific	Cat No. 17-5321-82, RRID:AB_469455
Brilliant Violet 605 anti-mouse IFN-γ	Biolegend	Cat No. 505840, RRID:AB_2734493
Alexa Fluor® 647 AffiniPure F(ab')$_2$ Fragment Goat Anti-Human IgG, F(ab')$_2$ fragment specific	Jackson ImmunoResearch Labs	Cat No. 109-606-006, RRID:AB_2337893
Anti-mouse IL-10 (Clone JES5-16E3), PE, eBioscience™	Thermo Fisher Scientific	Cat No. 12-7101-41, RRID:AB_10669561
Anti-mouse T-bet (Clone eBio4B10(4B10)), eFluor 660, eBioscience™	Thermo Fisher Scientific	Cat No. 50-5825-82, RRID:AB_10596655
Anti-mouse T-bet (Clone eBio4B10(4B10)), PerCP-Cyanine5.5, eBioscience™	Thermo Fisher Scientific	Cat No. 45-5825-82, RRID:AB_953657
Alexa Fluor® 488 Mouse anti-Bcl-6 (Clone K112-91)	BD Bioscience	Cat No. 561524, RRID:AB_10716202

PE Mouse anti-Bcl-6 (Clone K112-91)	BD Bioscience	Cat No. 561522, RRID:AB_10717126
Alexa Fluor® 647 Rat Anti-Blimp-1(Clone 6D3)	BD Bioscience	Cat No. 565002, RRID:AB_2739040
Anti-Mouse IgM (μ-chain specific)-Alkaline Phosphatase antibody produced in goat	Sigma-Aldrich	Cat No. A9688, RRID:AB_258472
Goat Anti-Mouse IgG, Human ads-AP	Southern Biotech	Cat No. 1030-04, RRID:AB_2794293
Goat Anti-Mouse IgG2b-AP	Southern Biotech	Cat No. 1091-04, RRID:AB_2794541
Chemicals, Peptides, and Recombinant Proteins		
Recombinant Mouse IL-21 R Fc Chimera Protein, CF	R&D	Cat No. 596-MR-100
Phorbol Myristate Acetate (PMA)	Sigma	Cat No. P1585
Ionomycin	Sigma	Cat No. I0634
Brefeldin A	Sigma	Cat No. B7651
Protein Transport Inhibitor (Containing Brefeldin A)	BD Bioscience	Cat No. 555029
Ricca Chemical Giemsa Stain	Fisher Scientific	Cat No. 3250-4
Mefloquine hydrochloride	Sigma	Cat No. M2319
Chloroquine diphosphate salt	Sigma	Cat No. C6628
CellTrace™ Violet Cell Proliferation Kit	Invitrogen™	Cat No. C34571
Experimental Models: Organisms/Strains		
C57BL/6J mice	The Jackson Laboratory	Cat No. 000664
B6.129S1-*Stat3*^{tm1Xyfu}/J (STAT3$^{fl/fl}$)	The Jackson Laboratory	Cat No. 016923
B6.129-*Prdm1*^{tm1Clme}/J (Blimp-1$^{fl/fl}$)	The Jackson Laboratory	Cat No. 008100
B6.129S6-*Tbx21*^{tm1Glm}/J	The Jackson Laboratory	Cat No. 004648
B6.Cg-Tg (CD4-Cre)1Cwi N9	Taconics	Cat No. 4196
Bcl6$^{fl/fl}$ x CD4-Cre	(Hollister et al., 2013)	N/A
Parasite Strains		
Plasmodium chabaudi chabaudi (AS)	Jean Langhorne, Crick Institute	N/A
Plasmodium yoelii (Clone 17XNL)	ATCC-BEI	
Software and Algorithms		
FlowJo™ (version 9.4.11)	FlowJo.LLC	https://www.flowjo.com/
FlowJo™ (version 10.5.3)	FlowJo.LLC	https://www.flowjo.com/
Prism	GraphPad	https://www.graphpad.com/scientific-software/prism/
SPICE (version 5.35)	NIAID-NIH	https://niaid.github.io/spice/

ELISA

Serum samples were obtained on the indicated days by bleeding mice from the tail vein under a heat lamp. Nunc-Immuno Plates (MaxiSorp™) were coated with whole freeze-thaw parasite lysate (transfer from N_2(l) to 37°C, 4-5 times (Guthmiller et al., 2017). Plates were blocked with 2.5% BSA + 5%FCS in PBS. Bound antibody was detected using Alkaline Phosphatase (AP)-conjugated goat anti-mouse IgM (Sigma), IgG and IgG2b (Southern Biotech, Brimingham, AL) which was revealed with a 4-Nitrophenyl phosphate disodium salt hexahydrate (PNPP, Sigma) solution (1 mg/ml). Plates were analyzed with a FLUOstar Omega plate reader (BMG Labtech, Cary, NC).

Statistics

Statistical analysis was performed in Prism (GraphPad, La Jolla, CA) using Student's *t*-test. $p <$ 0.05 was accepted as a statistically significant difference, * $p \leq 0.05$, **$p \leq 0.01$, ***$p \leq 0.001$, ****$p \leq 0.001$. Boolean gating analysis and Pie graphs were performed in SPICE software version 5.35 (http://exon.niaid.nih.gov/spice/).

Supplemental References

Hollister, K., Kusam, S., Wu, H., Clegg, N., Mondal, A., Sawant, D.V., and Dent, A.L. (2013). Insights into the role of Bcl6 in follicular Th cells using a new conditional mutant mouse model. J Immunol *191*, 3705-3711.
